# A Review on Viral Metagenomics in Extreme Environments

**DOI:** 10.3389/fmicb.2019.02403

**Published:** 2019-10-18

**Authors:** Sonia Dávila-Ramos, Hugo G. Castelán-Sánchez, Liliana Martínez-Ávila, María del Rayo Sánchez-Carbente, Raúl Peralta, Armando Hernández-Mendoza, Alan D. W. Dobson, Ramón A. Gonzalez, Nina Pastor, Ramón Alberto Batista-García

**Affiliations:** ^1^Centro de Investigación en Dinámica Celular, Instituto de Investigación en Ciencias Básicas y Aplicadas, Universidad Autónoma del Estado de Morelos, Cuernavaca, Mexico; ^2^Centro de Investigación en Biotecnología, Universidad Autónoma del Estado de Morelos, Cuernavaca, Mexico; ^3^School of Microbiology, University College Cork, Cork, Ireland; ^4^Environmental Research Institute, University College Cork, Cork, Ireland

**Keywords:** metagenomic, virosphere, extreme environment, viral gene bioprospection, extremophile virome

## Abstract

Viruses are the most abundant biological entities in the biosphere, and have the ability to infect Bacteria, Archaea, and Eukaryotes. The virome is estimated to be at least ten times more abundant than the microbiome with 10^7^ viruses per milliliter and 10^9^ viral particles per gram in marine waters and sediments or soils, respectively. Viruses represent a largely unexplored genetic diversity, having an important role in the genomic plasticity of their hosts. Moreover, they also play a significant role in the dynamics of microbial populations. In recent years, metagenomic approaches have gained increasing popularity in the study of environmental viromes, offering the possibility of extending our knowledge related to both virus diversity and their functional characterization. Extreme environments represent an interesting source of both microbiota and their virome due to their particular physicochemical conditions, such as very high or very low temperatures and >1 atm hydrostatic pressures, among others. Despite the fact that some progress has been made in our understanding of the ecology of the microbiota in these habitats, few metagenomic studies have described the viromes present in extreme ecosystems. Thus, limited advances have been made in our understanding of the virus community structure in extremophilic ecosystems, as well as in their biotechnological potential. In this review, we critically analyze recent progress in metagenomic based approaches to explore the viromes in extreme environments and we discuss the potential for new discoveries, as well as methodological challenges and perspectives.

## Introduction

Viruses are the most abundant biological entities in the planet, from the world oceans to the most extreme environments found in the biosphere (Zhang et al., 2018; Graham et al., 2019). Historically, the study of viral communities has been carried out by co-culture of viruses and their cellular hosts (Tennant et al., 2018), and more recently by viral metagenomic-based approaches (Nooij et al., 2018; Graham et al., 2019).

The exploration of viral populations in extreme environments has uncovered considerable genetic complexity and diversity. The biological organisms that inhabit extreme environments are termed extremophiles, and are found in all three domains of life (Merino et al., 2019). Like all other organisms, extremophiles serve as hosts for viral replication (Castelán-Sánchez et al., 2019). As viruses depend on a cellular host for replication, the interactions with their hosts affect microbial diversity, population interactions and dynamics, and even the genomes of these hosts (Le Romancer et al., 2006; Zhang et al., 2018; Castelán-Sánchez et al., 2019). In extreme environments their impact extends from influencing microbial evolution to playing an indirect but significant role in the earth’s biogeochemical cycles (Weitz and Wilhelm, 2012; Munson-McGee et al., 2018). However, despite their relevance, little is currently known about their ubiquity and diversity in extreme ecosystems (Paez-Espino et al., 2016; Berliner et al., 2018).

Nowadays the study of viruses can be carried out using metagenomic-based strategies that do not depend on cell culture approaches (Rose et al., 2016; Nooij et al., 2018; Zhang et al., 2018; Graham et al., 2019). Metagenomics represent a unique opportunity to describe the composition of viral communities in extreme environments, as well as to analyze the viral genetic reservoirs to characterize novel proteins and bioactive compounds of potential biotechnological utility.

Metagenomic studies are providing new sequences that in many cases do not share homology with sequences deposited in the reference databases (Hayes et al., 2017; Cantalupo and Pipas, 2019; Kinsella et al., 2019). It is evident that metagenomics from extreme habitats could be a powerful method to drastically increase the number of virus reported to date. It is surprising that despite the recent rapid advances in high-throughput sequencing techniques, there are still quite a limited number of studies describing the viromes of extreme environments in the literature. Here, we critically analyze recent progress in metagenomic-based approaches to explore the viromes in extreme environments, as well as methodological challenges and perspectives.

## Perspectives on Sampling and Processing: Methodological Challenges for Viral Metagenomics in Extreme Environments

Viral metagenomic studies are dependent on the ability to obtain sufficient amounts of nucleic acids from complex mixtures (Roux et al., 2019), particularly in extreme environmental samples that are as diverse as hot springs (Schoenfeld et al., 2008; Zablocki et al., 2017b), deep seawater, marine-sediments and oceanic basement (Breitbart et al., 2004; Hurwitz et al., 2013; Nigro et al., 2017), Antarctic and desert soils (Zablocki et al., 2014, 2017a), among others, to facilitate either the construction of metagenomic libraries or to perform direct sequencing.

The number of viral particles estimated to be present in a liter of water or kilogram of soil is in the order of 10^9^–10^11^, while the world’s oceans are estimated to contain up to 10^6^ viral particles per ml (Hara et al., 1991; Mokili et al., 2012). In contrast, the viral abundance from Octopus hot spring water from Yellowstone National Park or oceanic basement samples are at the lower range of ∼10^4^ and ∼10^5^ viral particles per ml respectively, compared with non-extreme aquatic environments (Schoenfeld et al., 2008; Nigro et al., 2017). In spite of viral particle abundance, during purification only subnanograms of viral DNA or RNA are typically recovered (Van Etten et al., 2010). Considering that a phage contains ∼10^–17^ g of DNA per particle, obtaining the amount of 1–5 μg of DNA required for standard pyrosequencing would implicate that ∼3 × 10^11^ viral particles should be recovered (Thurber et al., 2009), and even for third generation PacBio technology 10 μg of DNA is required (Faino et al., 2015). With the development of new sequencing technologies it is likely that lower amounts of nucleic acids will be needed. For example, only 50 ng of DNA was required to sequence bacteriophages and archaeal viruses from hypersaline environments (Motlagh et al., 2017; Liu et al., 2019) and surprisingly only 1 ng of DNA was needed to explore the metavirome from deep sea and the chaotropic salt lake Salar de Uyuni using KAPA Hyper Prep Kit and Nextera XT kits with Illumina platforms (Hirai et al., 2017; Ramos-Barbero et al., 2019).

### Filtration and Concentration of Viral Particles

To overcome the problem of obtaining sufficient viral nucleic acid amounts in extreme habitats, viral-particles must be concentrated by ultracentrifugation, flocculation, or filtration while minimizing contamination from prokaryotic or eukaryotic nucleic acids (Mokili et al., 2012; Liu et al., 2019; Ramos-Barbero et al., 2019; Roux et al., 2019). The use of classical size-selective ultrafiltration methods is not widely used, as the filters can often become blocked by impurities during concentration of the samples. Instead Tangential Flow Filtration (TFF) and/or ultracentrifugation were preferentially used in samples from hot springs and chaotropic salt lake Salar de Uyuni (Diemer and Stedman, 2012; Zablocki et al., 2017b; Ramos-Barbero et al., 2019). An excellent review by Lawrence and Steward (2010) on centrifugation highlights the efficiency of the methodology to sediment even the smallest viruses, where centrifugal separations can be divided in differential pelleting and zonal separations. The former has been successfully used to remove cell debris from samples obtained from enrichment cultures of archaeal viruses from an acidic hot spring Umi Jigoku in Beppu (Japan) (Liu et al., 2019) before concentrating and purifying viral particles and the latter has been tested using different gradient materials such as glycerol, OptiPrep and sucrose to isolate virus from a boreal lake in Finland (Laanto et al., 2017).

Due to the limitation of the volume size of aquatic samples, John et al. (2011) introduced the use of FeCl_3_ flocculation to concentrate viruses from seawater that results in the recovery of 92–95% of viruses, which has been favorably used in samples from glacier waters and deep-sea (Bellas et al., 2015; Poulos et al., 2018). This compares favorably with traditional centrifugation or TFF, which results in recovery levels of 18–26% and 62–93% respectively (Furtak et al., 2016), as evaluated by SYBR Gold staining, meaning that the use of FeCl_3_ increases the efficiency of viral particle recovery from extreme environment samples to around 30–60%.

A variation of the ultracentrifugation technique uses certain compounds to precipitate viruses like PolyEthylene Glycol (PEG) and ethanol, followed by purification through CsCl gradient ultracentrifugation (Fancello et al., 2013; Kleiner et al., 2015; Rastrojo and Alcamí, 2017; Chatterjee et al., 2019). These strategies have been implemented during viral recovery from Artic and Antarctic polar habitats, hot spring lakes and hypersaline lakes (Diemer and Stedman, 2012; Motlagh et al., 2017; Rastrojo and Alcamí, 2017; Mizuno et al., 2019).

Filters in the range of 0.1–0.22 μm have been used to enrich samples from South African hot springs and igneous crust of the seafloor (Nigro et al., 2017; Zablocki et al., 2017b). However, in recent years giant viruses –*giruses-* (particle size of ∼720 nm) have been discovered (Thurber et al., 2009; Van Etten et al., 2010) leading some groups to use 0.45 μm filters that are effective in recovering these larger viral-particles (Van Etten et al., 2010; Hurwitz et al., 2013; Sangwan et al., 2015). Up to now, limited knowledge about giant viruses in extreme niches has been produced. For example, a new large DNA virus named Medusavirus, was isolated from hot spring water in Japan using a filter of 1.2 μm (Yoshikawa et al., 2019). In addition, 64 members of the *Mimiviridae* family were recently identified in Antarctic marine water (Andrade et al., 2018). Thus, selective filtration strategies should be considered to recover extreme giant viruses.

Despite the use of a variety of different approaches to enrich viral particles from extreme environments, systematic studies comparing these different concentration methods (TFF, FeCl3, PEG, commercial concentrators) are still lacking and likely the methods employed for viral enrichment may need to be adapted considering the nature of the sample.

### Nuclease Treatment, Concentration and Viral Nucleic Acid Purification

Viral samples are usually treated with DNAse I, to avoid contamination with cellular genomic DNA that would, following sequence-based analysis, result in a large number of spurious DNA sequences from sources other than the virome. This treatment was used to obtain viral metagenomic DNA from Boiling Springs Lake (United States) and Great Salt Lake (thermophile and hypersaline ecosystems, respectively) (United States) (Diemer and Stedman, 2012; Motlagh et al., 2017). However, there are examples, such as in deep-sea ocean viral metagenomes (Hurwitz et al., 2013), desert perennial ponds (Fancello et al., 2013), hot springs (Zablocki et al., 2017b) among others, where despite the use of DNase treatment prior to viral genome purification, it was not possible to eliminate completely the cellular genome.

After DNase treatment, concentration steps are recommended using CsCl, sucrose or Cs_2_SO_4_ gradients by ultracentrifugation (Thurber et al., 2009; Fancello et al., 2013; Bellas et al., 2015). Additionally, once the viral particles have been concentrated and purified, the capsids have to be broken to release the viral genomes. The classical method is the use of formamide (Breitbart et al., 2002; Thurber et al., 2009; Fancello et al., 2013) followed by phenol:chloroform:isoamylic alcohol extraction (Diemer and Stedman, 2012; Nigro et al., 2017) or alternatively, through thermal shock (Bellas et al., 2015; Roux et al., 2016; Motlagh et al., 2017). However, in samples from hypersaline ponds, thermal shock may not be fully efficient to denude enveloped viral DNA, which may be the reason why in some studies the majority of viral DNA has been recovered from non-enveloped tailed viruses (Roux et al., 2016). Some single-stranded DNA (ssDNA) extreme viruses (e.g., HaloRubrum Pleomorphic ssDNA Virus 1, Haloarcula Hispanica Pleomorphic Virus 3, Aeropyrum Coil-shaped Virus) infecting hyperhalophile or hyperthermophile archaeal hosts, present a lipid envelope and multiprotein complexes or two criss-crossed halves of a circular nucleoprotein (Pietilä et al., 2010; Mochizuki et al., 2012; Demina et al., 2016) that could confer resistance to capsid disassembly. Thus, the capsid composition of extremophile viruses is a relevant feature of unknown viruses, as well as to access their genetic material, and consequently limits the identification of unusual extreme morphotypes.

### Retrotranscription or Amplification Steps

The identification of viral genomes to date has mainly focused on ssDNA or double-stranded DNA (dsDNA) viruses and only small RNA genomes of 5–10 kb have been assembled from extreme metaviromes. For example, RNA viruses infecting archaea were discovered from an acidic hot spring in Yellowstone (United States) (Bolduc et al., 2012; Wang et al., 2015), as well as from alkaline hot springs (Schoenfeld et al., 2008). In addition, RNA cyanophages have been recently reported from Porcelana hot spring in Chilean Patagonia (Guajardo-Leiva et al., 2018).

Andrews-Pfannkoch et al. (2010) have implemented the use of hydroxyapatite chromatography to efficiently fractionate dsDNA, ssDNA, dsRNA, and ssRNA genomes of known bacteriophages from samples of marine environments. This methodology has been employed to study ssDNA viruses from deep-sea sediments, alkaline siliceous hot springs and Artic shelf seafloor (Yoshida et al., 2013; Nguyen and Landfald, 2015; Schoenfeld et al., 2015), but to our knowledge it has not been applied to study RNA viruses from extreme ecosystems.

When working with RNA viruses, a retro-transcription step is required previous to library preparation, and if the efficiency of the nucleic acid recovery is low, amplification strategies are required. Among these, phi29 polymerase-based multiple displacement amplification and random PCR using modified versions of Sequence Independent Single-Primer Amplification (SISPA) have been useful in the virome amplification in samples from hot acidic lakes, hot springs and polar aquatic environments (Diemer and Stedman, 2012; Mead et al., 2017; Yau and Seth-Pasricha, 2019). When the viral genome material is RNA, a Random-Priming SISPA (RP-SISPA) method is frequently used (Miranda et al., 2016). This approach was successfully conducted in the isolation of RNA viruses from seawater (Steward et al., 2013) and Antarctic virioplankton (Miranda et al., 2016).

Other strategies for viral amplification are also used when extremophile metaviromes are studied. While the implementation of the Linker Amplified Shotgun Library methodology (LASL) is suggested to amplify dsDNA, Multiple Displacement Amplification (MDA) is employed to enrich ssDNA preferentially (Fancello et al., 2013). LASL has been performed to analyze viral metegenomes from Yellowstone hot springs and Antarctic virioplankton (Schoenfeld and Mead, 2015; Miranda et al., 2016), while MDA has been used to describe the virome present in deep-sea samples from Antarctica (Gong et al., 2018).

Zablocki et al. (2016) have argued that although viral amplification is commonly used in metavirome studies, especially for samples collected from extreme habitats such as hyperarid desert soils, this step should be avoided because it prevents the determination of viral particle abundance and diversity, and may promote a biased amplification of certain virus groups.

Thus, it is clear that further comparative methodological studies using samples from extreme environments are required to evaluate if purification, concentration and amplification methods have any impact in the virome structure obtained from metagenome analysis.

## Database and Bioinformatic Analysis: General Remarks

Up to now viral sequence search is conducted essentially on the NCBI database GenBank or RefSeq, according to their viral sequence classification criteria. The RefSeq database excludes some categories of data such as those that incorporate too much information that cannot be processed readily, such as metagenomes or genomes that have significant mismatch or indel variation compared to other closely related genomes. In addition, not all sequences have a taxonomic classification in the International Committee on Taxonomy of Viruses (ICTV) (O’Leary et al., 2015). The number of viral sequences reported in GenBank, reached almost two million by December 2018, of which only 3,279 were registered as genomes in RefSeq and of these, only 1,800 have a classification at the species level in the ICTV (Kang and Kim, 2018). The classification of viruses in ICTV has been based on the characteristics that can be used to distinguish one virus from another, such as the genome composition, the capsid structure, the gene expression program during viral replication, host range and pathogenicity, among others. Comparisons of both pairwise sequence similarity and phylogenetic relationships have become the primary guidelines used to define virus taxa (Simmonds, 2015). However, without the incorporation of metagenomic data in both the RefSeq and the ICTV databases, the comparison of sequences and their allocation is limited (Simmonds et al., 2017). Alternative viral sequence similarity search strategies, such as VirSorter (Roux et al., 2015) and VirFinder (Ren et al., 2017) have been developed. The former is designed to search protein-coding genes and the latter works with k-mer composition, both attempting to identify viral sequences in prokaryotic genomes. Integration of such strategies should reduce the number of unidentified sequences and the comparisons of viromes should then help to formulate more robust theories about their biological roles within a given community, thereby increasing the possibility of gaining a fuller understanding of the viromes in any environment (Simmonds et al., 2017).

**FIGURE 1:**
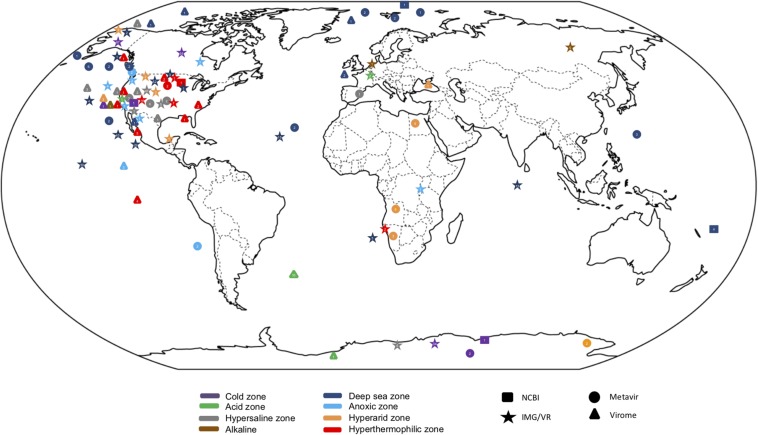
Global distribution of metagenomics studies from extreme environments from public databases. Circles represent metagenomes deposited in Metavir database, stars in IMG/VR, triangles in Virome and squares in NCBI.

Virus databases have been developed, such as Virome with 73 projects from viromes or metagenomes currently containing data from 270 libraries (Wommack et al., 2012); EBI metagenomics, a virome dataset and pipelines for analysis of metagenomes (Hunter et al., 2014); Metavir2, which is a web server for analysis of environmental viromes (Roux et al., 2014); and more recently, IMG/VR v.2.0 (Paez-Espino et al., 2018) that includes >600 extreme environmental metaviromes; Gut Virome Database (GVD) with 648 viral or microbial metagenomes (Gregory et al., 2019); iVirus (based on vConTACT as the main classification tool) which contains a dataset from 1,866 samples and 73 ocean expedition projects (Bolduc et al., 2017) (Figure 1). Some of them include viral sequences obtained through strategies such as the construction of fosmid libraries (Mizuno et al., 2013), cellular fraction of metagenomes (López-Pérez et al., 2017) or single viral genomics (Martinez-Hernandez et al., 2017), which enrich the virome sequences further. However, none of the above databases is particularly dedicated to viromes from extreme environments. Despite the limitations described above a comparative analysis of the population structure of viromes in extreme environments was carried out here, using publicly accessible virus metagenomic libraries as an attempt to exemplify the results that may be obtained using available tools and information. The data deposited in MetaVir2 until 2016 were selected because its user-friendly interface, which allows access to raw data or contig metagenome samples that contain well-classified metadata. We could select the 17 studies in MetaVir2 database that contain 66 viral metagenomes collected from most representative extreme environments: deep-sea (24), oxygen minimum zones (OMZ) (4), arid habitats (9), saline niches (23), cold environments (3) and hyperthermophile regions (3). The bioinformatic pipeline used was common to all data, so the comparison between environments relied on the same criteria. MetaVir2 followed two strategies to search the contigs in each sample: BLAST search in the RefSeq Virus database with the best-hit selection, and search for k-mer composition using di, tri or tetra nucleotides comparisons (Willner et al., 2009).

## Viromes in Extreme Environments

### Comparison of Viromes Between Extreme Habitats

The relative abundances from these data were analyzed comparing the similarities between environments. Their metadata are summarized in Supplementary Table S1.

**FIGURE 2:**
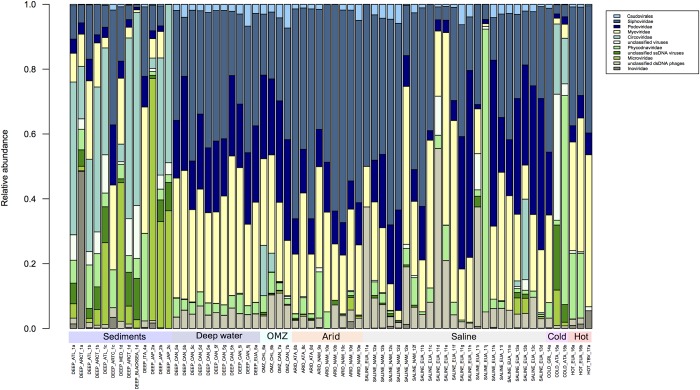
Comparison of the 10 most abundant virus families according to the Metavir database. The taxonomic composition is expressed in relative abundance at the virus family level. The families *Siphoviridae*, *Podovirididae*, and *Myoviridae* are ubiquitous in extreme environments. The figure was constructed from an abundance matrix, using the number of sequences reported in the Metavir database, from which the relative abundance was obtained; using the R program.

**FIGURE 3:**
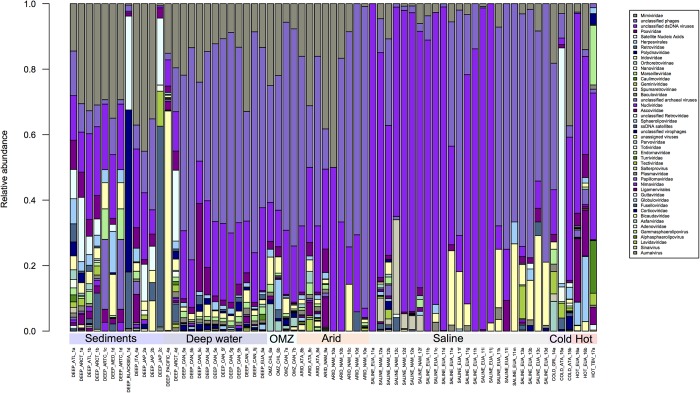
Comparison of families with lower abundance in the Metavir database. Unclassified phages predominate in all environments, compared with other virus families. However, greater diversity is observed in the sediments, hyperthermophile and hypersaline environments compared to deep waters, Oxygen Minimum Zones (OMZ) or saline environments.

The structure of the viral population in all the metagenomes analyzed by Metavir2 was compared (Figures 2, 3). The 10 most abundant families are represented in Figure 2 and the rest in Figure 3 to visualize the differences in abundance of each family. Some families of the order *Caudovirales* are ubiquitous and the most abundant were the *Siphoviridae*, *Myoviridae*, and *Podoviridae* as expected, since the viruses that belong to these families infect a wide range of bacterial hosts from more than 140 prokaryotic genera (Konstantinidis et al., 2009; Hurwitz et al., 2013; Danovaro et al., 2016; Graham et al., 2019). It has been considered that the information from whole metagenomic analysis can give clues of potential model microorganisms to host virus replication, through the analysis of the Clusters of Regularly Interspaced Short Palindromic Repeats (CRISPR) loci from the cellular fraction of the metagenomes, that have been isolated from extreme environments (Gudbergsdóttir et al., 2016; Sharma et al., 2018; Liu et al., 2019; Martin-Cuadrado et al., 2019).

Next in relative abundance were *Circoviridae*, followed by *Phycodnaviridae, Microviridae*, and *Inoviridae* and high fractions of unidentified ssDNA and dsDNA viruses and phages. *Circoviridae* infect vertebrates and were particularly abundant in sediment samples (Dennis et al., 2018, 2019; Blanc-Mathieu et al., 2019). Members of *Phycodnaviridae* have been found at high levels in deep water samples (Mizuno et al., 2016; Gong et al., 2018; Blanc-Mathieu et al., 2019), which is curious given that they preferentially infect eukaryotic algae which require light to grow. It is possible therefore that these dsDNA viruses may infect other as yet unknown marine hosts in deep waters (Van Etten et al., 2010; Blanc-Mathieu et al., 2019). However, the predominance of the above families present obvious exceptions, such as in two samples from cold environments, a sample from a saline environment and most of the samples from deep marine sediments.

Within the metagenomes that correspond to deep sea environments (depths greater than 1,000 m), where the absence of light, oligotrophic conditions, low-oxygen concentrations, low temperatures and high hydrostatic pressure dominate (Le Romancer et al., 2006; Liang et al., 2019), two categories were considered based on the origin of the samples: sediments and deep water (Figure 2). It is clear that in metagenomes from sediments of the Atlantic, Arctic and Pacific Northwest, ssDNA viruses like *Circoviridae*, *Microviridae* and *Inoviridae* are more abundant than dsDNA viruses (Figure 2). This characteristic seems to be exclusive to samples from this environment. It should be noted that the two samples from cold environments show a similar composition to that of sediments, and all others include ssDNA viruses in low abundance (Figure 2). This is in agreement with a recent report by Yoshida and coworkers who reported that ssDNA viruses predominate in marine sediments and have an estimated abundance of 1 × 10^8^ to 3 × 10^9^ genome copies per cm^3^ of sediment, clearly more abundant than dsDNA viruses which range from 3 × 10^6^ to 5 × 10^6^ genome copies per cm^3^ (Yoshida et al., 2018).

In Figure 3, where the remaining viral families are shown, two general points can be highlighted: *Mimiviridae* are present in almost all environments, which is not surprising since some of their hosts are known polyextremophiles (Claverie et al., 2018; Yau and Seth-Pasricha, 2019). The second point is the abundance of unclassified sequences, which do not allow any conclusion to be made about the diversity observed by environment since these sequences could come from one or more than one family. A large part of the sequences obtained from different environments, except for sediments, have no similarity in the databases, an issue that should change with the inclusion of additional metagenomic-derived sequences in databases (Figure 3). Overall analysis of the composition of viral families present in each extreme environment could at the very least allow a description of the families that are shared or that are exclusive to each environment.

Some environments are characterized by low-oxygen concentrations; these include those with high concentrations of greenhouse gases, which directly affect the biodiversity in those environments (Kiehl and Shields, 2005; Resplandy et al., 2018). There are three central oceanic regions which are considered to be Oxygen Minimum Zones (OMZ), namely the Eastern Tropical North Pacific (ETNP), the Eastern Tropical South Pacific (ETSP) and the Arabian Sea, within which the activity of anaerobic microorganisms is highly significant (Paulmier and Ruiz-Pino, 2009; Thamdrup, 2012). As expected, the viral population diversity closely reflects the microbial diversity in these environments (Cassman et al., 2012; Parvathi et al., 2018; Fuchsman et al., 2019), with the virome composition in OMZ being commonly composed of the *Myoviridae* and *Siphoviridae* families, followed by *Phycodnaviridae* (Figure 2).

OMZ were sampled at 200 m depths in Chile and Canada and virus composition was analyzed using the MDA (Genomiphi and GenomePlex) protocol. While the ssDNA *Circoviridae* family was predominantly observed in samples from Chile, this virus family was not observed in samples from the Canadian OMZ. In addition, in the samples from Canada (Chow et al., 2015) *Parvoviridae* (ssDNA) were highly abundant, but were totally absent in samples from Chile (Figure 3). In previous studies it was observed that the viral community along the vertical dissolved oxygen gradients was characterized by abundance taxa and diversity fluctuations. These differences could be related to changes in the viral replication strategy from lytic to lysogenic. It seems that oxygen reduction concurs with a decrease in viral abundance (Cassman et al., 2012; Parvathi et al., 2018). It should be noted that a large proportion of sequences obtained from these regions do not find similarity with other viruses in the databases, but those sequences could be from viruses that infect little known prokaryotic hosts, like ammonia-oxidizing archaea and anaerobic ammonia-oxidizing (anammox) bacteria which predominate in this environment (Parvathi et al., 2018).

Hyperarid environments exhibit conditions that are considered to be limiting for life, such as lack of water, high levels of UV radiation and extreme temperatures. However, both prokaryotic and eukaryotic organisms have adapted to live in these environments (Merino et al., 2019). Although low diversity might be expected in these environments, metagenomic studies performed with hypolithic communities have shown this not to be the case, with a high level of diversity being reported; particularly in bacterial communities from Antarctica (cold desert) and Namibia (desert), which are mainly *Actinobacteria*, *Proteobacteria*, and *Cyanobacteria* (Vikram et al., 2016). In the hypolithic viral communities from the Namibian desert and the Antarctic, metagenomic data has revealed the presence of *Caudovirales* which do not correlate with phages that infect *Cyanobacteria* species (Adriaenssens et al., 2015). The samples from the Antarctic hyperarid region displayed a greater diversity of unique viruses such as *Bicaudaviridae*, *Asfarviridae*, *Lavidaviridae*, *Tectiviridae*, and *Sphaerolipoviridae* when compared with the families found in the Namibian desert (Figure 3). Zablocki and coworkers have previously reported a higher viral diversity in the Arctic when compared with the Namibian desert, and it has been observed that Antarctic desert soils contain higher proportions of free extracellular virus-like particles compared to hot hyperarid desert soils, where a lysogenic lifestyle seems to prevail (Zablocki et al., 2016).

In Figure 3 the variability in the composition of viral families in hypersaline habitats is evident. Such environments are widely distributed throughout the world and are present in salt lakes, salt flats and salt deposits. In these environments, the low water activity directly affects the composition of the microbial communities (Le Romancer et al., 2006; Ma et al., 2010; Merino et al., 2019). Viruses that have been identified in these ecosystems are haloviruses and a large number of these infect Archaea, Bacteria and Eukaryotes (Atanasova et al., 2018; Plominsky et al., 2018; Ramos-Barbero et al., 2019). About 64 archaeal viruses have been isolated from the two kingdoms, *Crenarchaeota* and *Euryarchaeota* (Porter et al., 2007). These samples are also those that have a greater abundance in unassigned or not classified viruses, which prevents determination of the real diversity of that group of archaea viruses, probably because they are the least studied and have low representation in the databases (Atanasova et al., 2018; Ramos-Barbero et al., 2019).

In addition, unclassified dsDNA viruses have also been observed (Figure 3), while haloviruses such as HGV-1, HTVAV-4 and HSTV-1 have also been identified at high levels. On the other hand, ssDNA viruses which mostly infect eukaryotes such as colpodellids, nematodes, arthropods, chlorophytes, among others, are present at low levels in hypersaline habitats (Feazel et al., 2008; Heidelberg et al., 2013).

The thermophile environments are characterized by high temperatures, where thermophilic microorganisms thrive at 65–80°C as their optimal growth temperature, and >80°C for hyperthermophiles (Merino et al., 2019). Viruses that infect bacteria and archaea are abundant in these hyperthermophilic habitats (Schoenfeld et al., 2008; Strazzulli et al., 2017; Liu et al., 2019). The virome of hyperthermophile environments is composed of viruses that infect all three domains of life, with members of the *Turriviridae, Fuselloviridae, Bicaudaviridae*, and *Globuloviridae* families that infect Archaea (Krupovic et al., 2018). Moreover, the *Nudiviridae, Phycodnaviridae*, and *Poxviridae* families that infect eukaryotes are also present (Figure 3).

Within the hyperthermophile metagenomes analyzed here, the presence of ssDNA or RNA viruses was not observed, but in other studies from these environments the presence of picornavirus-like, alphavirus-like, and flavivirus-like RNA viruses has been reported (Bolduc et al., 2012). It is possible that ssDNA and RNA viruses were not detected in the samples we analyzed due to differences in sample processing (Figures 2, 3). Thus, as previously mentioned, if comparative viral metagenomic studies are to be undertaken to allow an accurate comparison between viromes from different ecosystems and to potentially identify novel viral clusters, then standardized methodologies will need to be developed and employed.

The polar regions of the Earth are dominated by the polar ice caps, with the microbial diversity present in these regions being much higher than might be expected. It is well established that viruses play an important role in controlling microbial mortality in these habitats (López-Bueno et al., 2009; Cárcer et al., 2015; Yau and Seth-Pasricha, 2019). While it has been reported that different lakes located in the Arctic and Antarctic share similar virome compositions, marked differences have been found.

Although at this taxonomic level it is possible to differentiate some of the particularities described above, in terms of the virus composition in each environment studied, very little information is revealed at the genus or species level that would allow a better understanding of the virus–host relationship and its influence in the environment.

**FIGURE 4:**
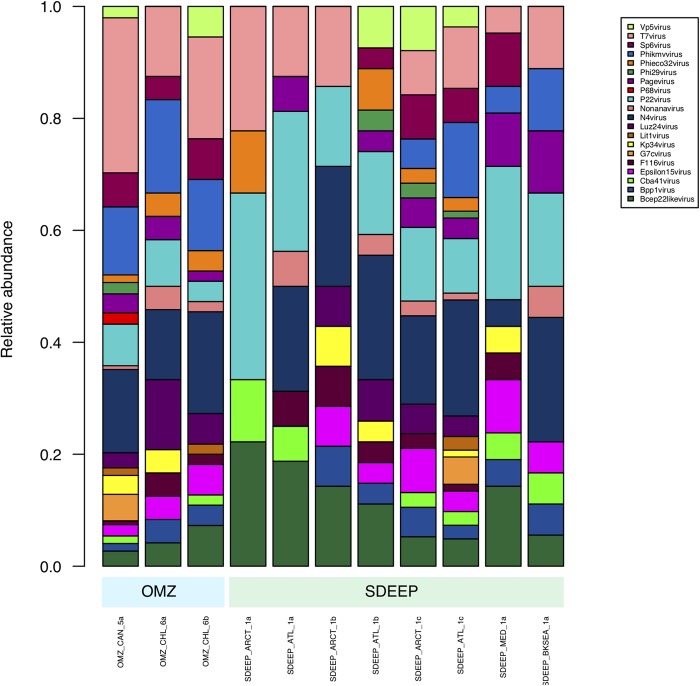
Composition of *Podoviridae* family at genus level. Metagenomes from OMZ and deep-sediments were considered in the analysis.

**FIGURE 5:**
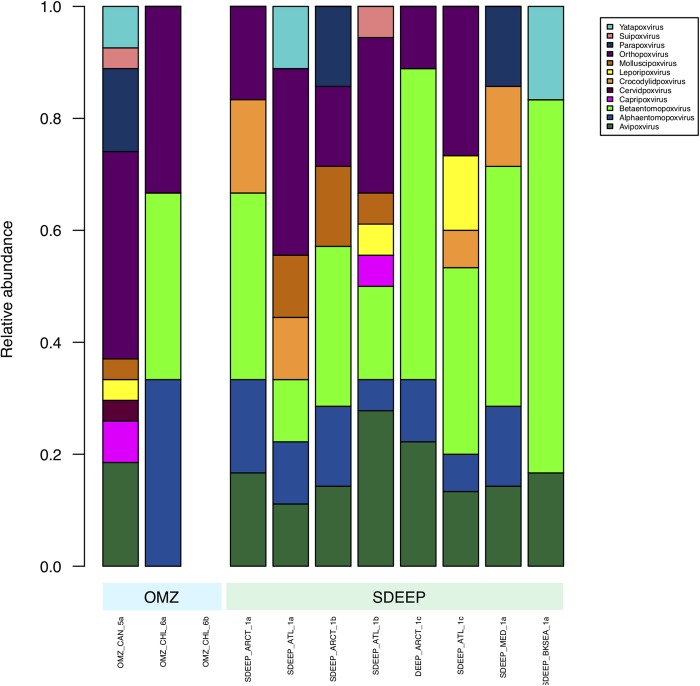
Composition of *Poxviridae* family at genus level. Metagenomes from OMZ and deep-sediments were considered in the analysis.

Therefore, two environments, OMZ and deep-sediments, which at the family level have a very similar structure (Figures 2, 3) were selected in an attempt to determine if it is possible to obtain biologically meaningful information on the differences or similarities in virus-host interactions at the genus level. The genus composition of two well-known families were analyzed: *Podoviridae* that infect bacteria and are ubiquitous even in extreme environments (Figure 4), and *Poxviridae* (Figure 5) for which known hosts are terrestrial vertebrates and invertebrates and their presence in extreme environments, particularly in aquatic environments has not been reported. At this level only some viruses can be taxonomically identified and can be seen to vary in abundance, however, most of the genera were unidentified. The genera from *Podoviridae* identified by sequence were enterobacter phages, which infect bacteria that are known human pathogens, and would not be expected in these environments (Figure 4). In the case of *Poxviridae*, most genera found infect terrestrial vertebrates, suggesting that either the sequences of the *Poxviridae* family members obtained from aquatic niches are sufficiently similar to those from *Poxviridae* members that infect terrestrial hosts, or this is an artifact caused by the lack of sequences from *Poxviridae* found in the databases (Figure 5).

### Clustering by Environmental Virome

**FIGURE 6:**
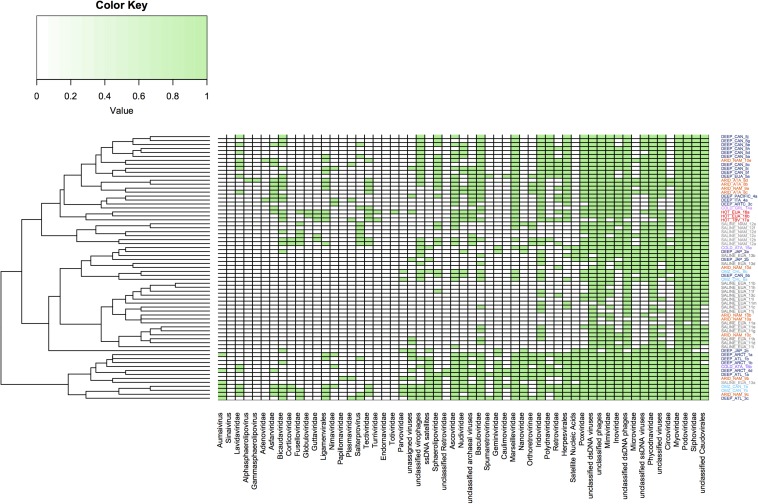
Clustering dendrogram based on the abundance of virus families present in the metagenomes from extreme environments. Virus families are represented by presence or absence; the green color indicates the presence while the white color indicates absence. Families of the order of Caudovirales are present in all environments. The figure was constructed using a presence and absence matrix, using the heatmap library on R.

Hierarchical clustering analysis was performed from abundant viral families previously published in the aforementioned metagenomic datasets (Figure 6). From this it was possible to conclude that some extreme environments have groups that indicate similarities in the viral communities present in these environments. This was particularly evident for some viromes obtained from hypersaline, deep-sea and hyperarid environments, while it was less evident in other extreme ecosystems which did not appear to show clustering, such as cold environments. However, this analysis again shows that some viral families are ubiquitous in all extreme environments, while the ssDNA viruses appear to predominate in sediments from deep-sea and cold environments.

In general, the virome structure from hypersaline samples reveals low levels of diversity, even in samples from different geographical areas (Figure 6). The high concentration of NaCl might limit viral diversity due to the shortage of prokaryotic hosts, since *Haloquadratum walsbyi*, *Salinibacter ruber*, and nanohaloarchaeas are the predominant organisms in these environments, with more than 90% of the contigs annotated to these taxa (Ventosa et al., 2015). Another factor that could determine the virome diversity that is observed in hypersaline environments is the dynamic switch between lytic and lysogenic replication cycles, since this represents a significant adaptation mechanism in environments with high salinity content (Roux et al., 2016).

This notwithstanding, from Figure 6 it is clear that the virus family composition is quite similar in these environments, which could provide significant information related not only to viral evolution but also to physiological adaptation of microorganisms in response to high temperatures (Schoenfeld et al., 2008; Biddle et al., 2011).

**FIGURE 7:**
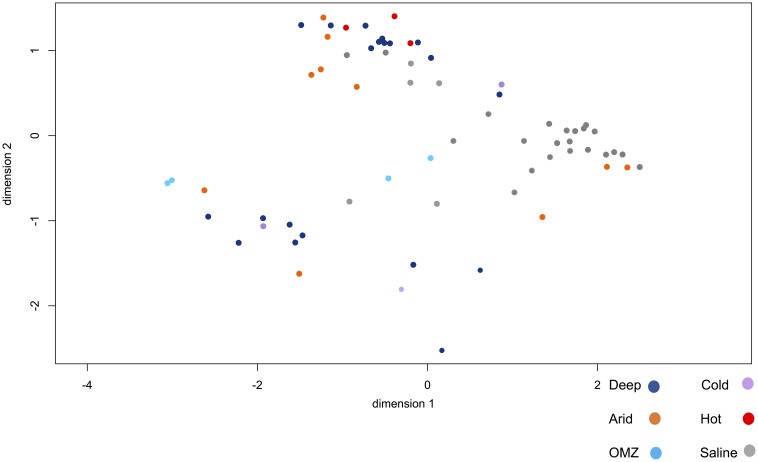
Multidimensional scaling (NMDS) to visualize the degree of overall similarity between the metagenomes. Metagenomes from different habitats are shown: deep-sea (pink), OMZ (green), hyperarid (red), hypersaline (blue), psychrophile (orange), hyperthermophile (yellow). Hypersaline metagenomes have a strong clustering compared to other metagenomes. The figure was constructed from an abundance matrix, where the multidimensional scaling algorithm was applied to observe the general similarity of metagenomes, using the R software.

Figure 7 shows the degree of overall similarity between the viral metagenomes in relation to the extreme environment from which the viromes were isolated. As previously described, some viral families belonging to the *Caudovirales* order are ubiquitous and display polyextremophilic adaptation. The hypersaline environments present a consistent clustering depending on the viral diversity, as well as the relative viral family abundance, which suggests that NaCl enriched environments provide strong constraints for the development of life that may restrict ecosystem diversity. Some viromes from hyperarid, deep-sea and saline environments are closely clustered (Figure 7) suggesting that the organisms and therefore the viral composition is partially shared, at least between these environments. Regarding deep-sea environments, Figure 7 shows two clustered metagenome populations derived from the deep-sea, where those viromes obtained from deep water are closely clustered, as well as those from sediments.

An interesting hypothesis to be investigated using metagenomics studies conducted in different geographical areas is the possibility of identifying specific viral clusters associated with a particular extreme environment. The large numbers of unclassified sequences in the databases is an important issue to consider with studies on viromes from extreme environments. The limitations of the bioinformatic pipelines to assign a taxonomic identity to a majority of the viral sequences, together with our limited understanding about viruses in extreme environments, has resulted in a lack of progress in our knowledge of extremophilic viromes. This has also negatively impacted our understanding in terms of evolution, gene horizontal transfer, ecology and virus-host interactions.

To meaningfully compare viromes from different environments it is necessary to at least partially answer the previous questions and provide new information about how viromes are potentially limited by extreme physicochemical characteristics, geographical area, or other artificial circumstances such as sampling methods, enrichment techniques and other technical biases. It should be possible to determine whether some viral populations could be closely related to a specific type of extreme ecosystem and consequently obtain more information about viral evolution (Simmonds, 2015).

## Functional Metagenomics in Extreme Environments: Methodological Challenges, Discoveries and Opportunities

**FIGURE 8:**
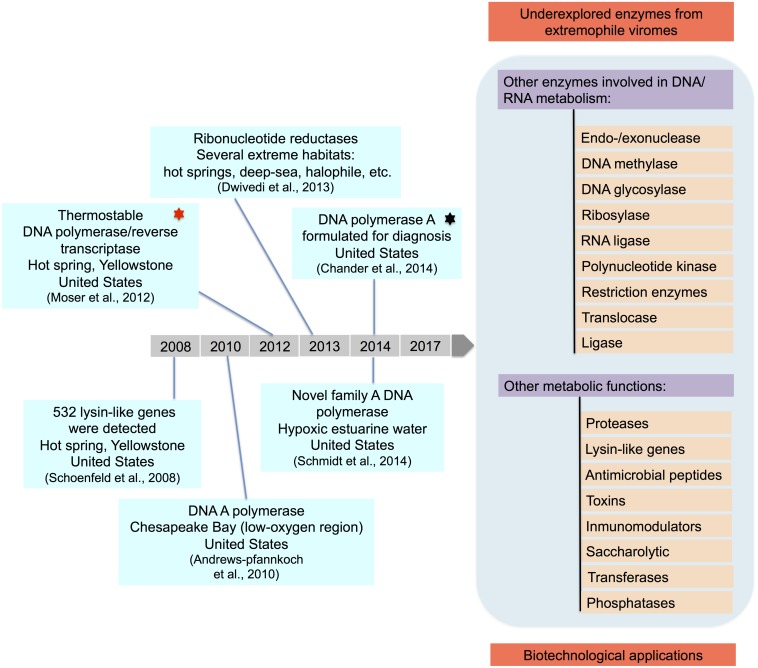
Progress in function-based metagenomics. Functional bioprospections from extreme environments performed in the last years are summarized. Underexplored enzymes from extremophile viromes are listed.

Functional viral metagenomics focuses on exploring viral diversity to discover novel genes. Extreme environments harbor an enormous diversity of unknown viruses (Desnues et al., 2008; Dinsdale et al., 2008; Williamson et al., 2008; Rosario and Breitbart, 2011; Kristensen et al., 2012; Atanasova et al., 2016; Gudbergsdóttir et al., 2016; Nigro et al., 2017; Zablocki et al., 2017a, b; Sharma et al., 2018; Liu et al., 2019; Martin-Cuadrado et al., 2019; Mizuno et al., 2019; Roux et al., 2019) and, consequently, a potentially large number of unknown viral proteins. Functional viral metagenomics in these niches show a limited progress, with few reported recent advances (Schoenfeld et al., 2009; Schmitz et al., 2010; Moser et al., 2012; Heller et al., 2019) (Figure 8).

Viral-host systems (*in vitro* screening), sequence-based screening, activity-based screening (heterologous expression of viral proteins), and PCR- and hybridization-based screening, could be implemented for functional analysis from extreme viromes (Moser et al., 2012; Bzhalava and Dillner, 2013; Fancello et al., 2013; Heller et al., 2019). While sequence-based screenings have been responsible for the discovery of the majority of new viral enzymes (at least as annotated proteins) from extreme environments, PCR- and hybridization-driven methods have not been employed to date to functionally explore extremophile viromes.

*In vitro* screening from extremophile viromes is a challenge with respect to the co-cultivation of both hosts and viruses, and in particular in trying to mimic the conditions which are present in these habitats, thereby ensuring a better success rate regarding viral replication and viral protein expression. Thus, to help overcome this bottleneck, it will be important to develop new host systems (for prokaryotic and eukaryotic viruses) which grow under extreme pH, temperature, salinity, pressure and radiation (Schoenfeld et al., 2009).

Activity-based screening could be a very useful approach to identify novel enzymes. This method demands an efficient heterologous expression system for viral proteins. Thus, there are problems with the expression levels of many viral proteins in foreign host systems, particularly in genes isolated from extremophile viromes, which are dominated by rare genes, with issues such as codon usage together with promoter regulation/activation negatively impacting on enzyme production in different heterologous systems (Kristensen et al., 2012).

While the well-established *Escherichia coli* heterologous expression system is available, it is clear that additional systems with a particular focus on extremophile bacteria and fungi will need to be developed to increase the chances of producing sufficient levels of viral extremoenzymes to allow their detection in function-based screens. These screens usually employ activity-based assays which involve colorimetric changes, typically following utilization of a substrate. However, these types of screenings are not particularly useful with viromes, since viral genes encoding enzymes involved in the metabolism of different substrates are quite rare. Due to this, there are no reports to date of the detection of viral enzymes from viromes through activity-based screening.

Despite the aforementioned disadvantages related to the heterologous expression of viral proteins, the activity-driven screenings allow functional gene annotation through an *in vitro* phenotypic-based test. This has an important advantage over sequencing-driven screening where a high number of genes are annotated as “*unknown function,”* because the gene repertoires of the extremophile viromes are currently undersampled.

A number of viral enzymes with utility in scientific, diagnosis and therapeutic applications have been identified using sequencing-based screens from extremophile viromes (Schoenfeld et al., 2008, 2009; Moser et al., 2012; Dwivedi et al., 2013; Schmidt et al., 2014; Mead et al., 2017). Also, the genomes of extremophile viruses are likely to be a source of novel antimicrobial peptides that may have applications in the biopharmaceutical and molecular diagnostics areas (Rice et al., 2001; Le Romancer et al., 2006; Schoenfeld et al., 2009).

For example, a sequencing-driven metagenomic study from two mildly alkaline hot springs in Yellowstone, allowed identification of 532 lysin-like genes (Schoenfeld et al., 2008). In recent years, these lytic enzymes have gained increasing importance due to their potential use in biomedical science applications (Schmitz et al., 2010); however, no lysin-like genes from extreme environments have to date been experimentally characterized.

DNA polymerases (502 sequences) have been also detected in extreme metaviromes, particularly from hypoxic estuarine waters obtained in the Gulf of Maine, Dry Tortugas National Park and the Chesapeake Bay (Andrews-Pfannkoch et al., 2010; Schmidt et al., 2014). These shotgun metagenomic studies revealed a novel DNA polymerase A family in marine virioplankton, since some sequences were distantly grouped in a phylogeny comprising DNA polymerase A from virus and bacteria (Schmidt et al., 2014).

Ribonucleotide reductases (RNR) have also been found from viromes obtained from hypersaline, psychrophile and thermophile niches (Dwivedi et al., 2013). For example, a bioinformatics analysis demonstrated that viruses isolated from hot springs contained a high abundance of RNR. However, some habitats such as hydrothermal vents from the East Pacific Rise, a solar saltern pond and salterns from Alicante (Spain) were found to have fewer (≤5) identifiable RNR viral homologs (Dwivedi et al., 2013).

Recent efforts to characterize new viral DNA polymerases from extreme environments have resulted in the identification of a thermostable polymerase in a viral metagenomic DNA library from a near-boiling thermal pool in a hot spring in Yellowstone (Moser et al., 2012; Heller et al., 2019). This was the first report describing the isolation of a polymerase from a viral metagenomic library. In this study 59 complete polymerase clones were identified as possessing thermostable DNA polymerase activity following a functional screen. One of these polymerases, namely PyroPhage 3173 Pol, also has 5′-3′ exonuclease activity, as well an innate reverse transcriptase activity. It was subsequently tested in high fidelity reverse transcription PCR (RT-PCR) reactions and compared with some commercially available enzyme systems (Moser et al., 2012; Heller et al., 2019). The PyroPhage 3173 Pol-based RT-PCR enzyme was found to have a higher specificity and sensitivity that the other enzymes. While the PyroPhage 3173 DNA polymerase shares amino acid identity (∼32%) with another bacterial polymerase, no significant similarity was found with other viral proteins (Moser et al., 2012). This highlights the potential diversity of enzymes that may be present in extremophile viromes. The enzyme has subsequently been characterized and shown to be effective in the molecular detection of certain viral and bacterial pathogens by loop-mediated isothermal amplification (Chander et al., 2014).

## Structural Biology of Viromes

Specific molecular-level adaptations to extreme environments can only be appreciated once the detailed molecular structures are known. In order to explore the available structural information of proteins belonging to extremophile viruses, we carried out a manual search in the Protein Data Bank^1^ (Berman et al., 2000) of all the viral families and genera identified in the metagenomes analyzed in this review, and kept only those whose hosts were either marine viruses or frank extremophiles. The resulting proteins were then classified according to their annotated function, and are discussed below.

In general, all these structures are valuable from a biochemical and biotechnological perspective, as they contain the molecular representation of the required adaptation to the particular extreme environment favored by the virus host. For example, viruses that infect *Acidianus* or *Sulfolobus* archaea are subject to the combination of high temperature and acidic pH; their proteins tend to have many charged residues, in particular, acidic ones (see structure 3DF6, an orphan protein). They also tend to have compact folds with structured termini, short loops with prolines in specific positions to stabilize them (see structure 2BBD, a major capsid protein), and the absence of cavities. Despite being DNA-binding proteins, and therefore cytoplasmic, some of them also include disulfide bridges, intramolecular (see structure 2VQC) or intermolecular (in structure 2CO5), that can impart up to 14 degrees in thermal stability for the protein. The formation of these disulfide bonds requires the existence of a sulfhydryl oxidase, either encoded by the host or by the virus itself. When a mesophilic homolog exists, a direct comparison of the structural features of the proteins can guide protein engineering to improve stability and/or function.

Also, as some viruses have space limitations in their capsids, resulting in compact genomes, viral homologs in these cases tend to be the minimal possible version of the protein family, allowing for the identification of the critical residues that stabilize both structure and function. A nice example of this is the minimal catalytic integrase domain of *Sulfolobus* spindle-shaped virus 1 (structures 3VCF, 4DKS and 3UXU). On the other hand, for viruses that have less space limitations, viral proteins can have surprising combinations of domains, suggesting ways to engineer multidomain proteins. This is particularly notorious in *Mimivirus*, where identifiable catalytic domains can be linked to domains with no sequence or structure homology to any known protein, as in the sulfhydryl oxidase in structure 3TD7. Less dramatic examples are basic modules known to function as transcription factors, such as the ribbon-helix-helix domain, with an extra helix added as an embellishment that increases thermal stability, as in structure 4AAI from *Sulfolobus* virus Ragged Hills.

The analysis of the conservation of proteins amongst viruses of the same or different classes is instructive, and can help in establishing families and/or events of horizontal gene transfer. This conservation has been historically one of the criteria used to choose which proteins to study structurally from a particular virus. The wealth of information derived from identifying open reading frames in the data from sequencing endeavors can certainly be a source of novel activities, as described in the previous section. This functional annotation requires sequence homology to known proteins, something that does not happen frequently with extremophile viruses. As structure diverges more slowly than sequence, protein structural analysis allows for the inference of function when sequence homology is weak. In Supplementary Table S2 we list viral protein structures that were obtained in this spirit, sometimes as part of Structural Genomics Initiatives (Oke et al., 2010). As can be seen from Supplementary Table S2, the goal of assigning function is not always achieved, as on occasion novel folds are found (see, for example, structures 4ART and 3DF6 discussed above), precluding the transfer of function. In other, happier cases, the structure instructs the experiments needed to functionally annotate the sequence (see structure 3O27, with functional DNA binding activity).

Most of the structures we found were obtained with a previous inkling of the function of the protein. For example, Supplementary Table S3 lists structural proteins, such as capsids and tail spikes. The full capsid structures are interesting, for instance, as scaffolds for drug delivery, and as models to study capsid formation, propose infection mechanisms, and study the interactions with nucleic acids and membranes. In this regard, structure 5W7G proposes a model for the membrane envelope of *Acidianus* filamentous virus 1, composed of flexible tetraether lipids that are organized as horseshoes, including a mechanism for enrichment of the viral membrane with this particular lipid of low abundance in the host. Another important interaction is that of capsid proteins with DNA, and surprisingly, it appears that rod-shaped viruses (such as *Sulfolobus islandicus* rod-shaped virus 2 in structure 3J9X) organize their DNA in the A form, stabilized by alpha helices from the major capsid proteins. This is in stark contrast to icosahedral viruses, which pack their DNA in the B form. Turrets, tails and spikes are important to understand interactions with the hosts, as part of the ecological role that these viruses play.

Supplementary Table S4 lists proteins that bind either DNA or histones; the latter come from viruses that infect either fish or shrimp and are interesting because one of them is a DNA mimic (see structure 2ZUG). The architecture of these DNA-binding proteins is sometimes reminiscent of known bacterial classes (see structure 2CO5, a winged helix-turn-helix protein with an intramolecular disulfide bond, discussed above), or is a novel fold (see structure 2J85). Finally, Supplementary Table S5 lists enzymes found in extremophile viruses. The range of activities is wide, going from DNA, protein and sugar metabolism, to reactive oxygen species management (see, for example, structure 4U4I, a superoxide dismutase that does not require chaperones to capture copper or oxidize its disulfide bridges). There is also interest in auxiliary metabolic genes that support more efficient phage replication, and are normally related to photosynthesis; in this class we find structure 5HI8, a phycobiliprotein lyase, and 3UWA, a peptide deformylase particularly selective for the D1 protein of photosystem II.

The relevance and utility of all these structures is multiple: as crystallographic-amenable homologs of difficult targets (see structure 3VK7, a DNA glycosylase) given their stability, as inspiration to improve mesophilic orthologs in their resistance to high temperature and low pH, as examples on how to trim these orthologs to minimal yet functional versions, in the identification of novel quaternary structures (see for example structure 5Y5O, a dUTPase with novel packing), and as examples on how to adapt new modules to them (see structure 3TD7, the sulfhydryl oxidase with a novel domain attached at the C-terminus). The field of structural biology of extremophile viruses is still young, and there is plenty of room for the exploration of orphan ORFs and for viruses subject to other extreme environments.

## Final Remarks

Metagenomics is a powerful approach to study the virome structure of extreme environments and its potential biotechnological applications in a number of fields. However, despite its potential few studies have been undertaken to characterize viral communities in these environments. Some methodological challenges need to be overcome to ensure that samples enriched in viral particles can be obtained, as well as increasing the yields of viral nucleic acids that can be isolated.

A comparative analysis of the population structure of viromes in extreme environments was carried out here, using the 17 publicly accessible virus metagenomic libraries deposited in MetaVir2. Viral communities from different extreme environments showed quite high levels of overall similarity, with viral families belonging to the *Caudovirales* order being ubiquitous and displaying seemingly polyextremophilic adaptation. The most abundant families of *Caudovirales* were *Siphoviridae*, *Myoviridae*, and *Podoviridae*, followed by *Circoviridae, Phycodnaviridae, Microviridae*, and *Inoviridae*. However, very high fractions of unidentified ssDNA and dsDNA viruses and phages were identified. Considering the large number of unclassified viral sequences from extremophilic viromes, it is currently not possible to definitively identify novel virus families which are uniquely present in different extreme environments. Nor is it possible to correlate the presence of specific virus families or genera with any given environment.

Attempts to further explore specific virus–host relationships in the *Podoviridae* and *Poxviridae* present in OMZ and deep-sediments resulted in the identification of viruses whose known hosts are highly unlikely to reside in these environments. Although many more sequences were obtained when a similar comparison was made using newer, more up to date databases (RefSeq from NCBI, or IMG/VR), the viruses identified often correspond to those for which more sequences are available. Therefore, although MetaVir2 is no longer kept up to date, and richer, more recent databases exist, such as IMG/VR, a similar comparative analysis using such databases produced similar results (not shown), indicating that more accurate taxonomic assignments are required and ideally they should be in a common repository where all the viral metadata are collected. Also new tools should be developed to automatize sequence classification so that viral species assignment can be obtained.

Hierarchical clustering analysis was performed from abundant viral families previously mentioned and it was possible to conclude that some extreme environments, such as hypersaline, deep-sea and hyperarid niches, have groups that indicate similarities in the viral communities present in these environments, although as above, with the available data this comparison could only be evaluated at the family level. An important challenge from viral metagenomics is to establish specific viral clusters associated with particular extreme environments and describe their role in different extreme ecosystems. Although taxonomic allocation at the level of the genus or species in viruses is a challenge, new strategies for the classification of viruses are still in development from the use of genomic sequences without previous information or clustering of their coding sequences, that allow a more efficient classification process, that is scalable and user friendly.

In addition, the functional prospecting for viruses in extreme ecological niches has been almost exclusively limited to sequence-based screening to date. While some viral sequences have been annotated and assigned to specific functions, very few viral proteins discovered using metagenomics approaches have been subsequently cloned, heterologously expressed and biochemically characterized. Other functional-based methods such as activity-based screenings and PCR -or hybridization- based screenings are currently underexploited as approaches to identify viral proteins from extremophilic viromes, which may have utility in biotechnological applications. Considering that our current knowledge of viromes associated with extreme ecosystems is quite limited, we still cannot fully appreciate the great biotechnological potential that they may represent. Thus, further efforts should be made to screen extremophile viral metagenomes for novel proteins and biomolecules if we are to advance our understanding of their biological impact and to capitalize on the unique viral diversity that is present within these novel ecosystems.

## Author Contributions

SD-R, RG, AD, and RB-G designed and wrote the manuscript. MS-C prepared the Section “Perspectives on Sampling and Processing: Methodological Challenges for Viral Metagenomics in Extreme Environments.” HC-S, SD-R, RP, and AH collected and processed the metagenomic data and conducted the analysis of viral communities. RB-G and LM-Á prepared Section “Functional Metagenomics in Extreme Environments: Methodological Challenges, Discoveries and Opportunities” related to functional bioprospection in extreme viromes, while NP prepared Section “Structural Biology of Viromes.”

## Conflict of Interest

The authors declare that the research was conducted in the absence of any commercial or financial relationships that could be construed as a potential conflict of interest.

## Footnotes

1 rcsb.org
