# Supplementary material for: A Review on Viral Metagenomics in Extreme Environments
Source: Front Microbiol. 2019 Oct 18;10:2403. doi: 10.3389/fmicb.2019.02403 (PMC6842933; doi:10.3389/fmicb.2019.02403)
Supplement: Supplementary file 1 [file Data_Sheet_1.PDF]

A review on viral metagenomics in extreme environments

Running title: Viral metagenomics in extreme environments

Sonia Dávila-Ramos[1], Hugo G. Castelán-Sánchez[1], Liliana Martínez-Ávila[1], María del Rayo Sánchez-Carbente[2], Raúl Peralta[1], Armando Hernández[1], Alan D.W. Dobson[3,4], Ramón A. Gonzalez[1], Nina Pastor[1], Ramón Alberto Batista-García[1*]

[1]Centro de Investigación en Dinámica Celular. Instituto de Investigación en Ciencias Básicas y Aplicadas. Universidad Autónoma del Estado de Morelos. Cuernavaca, Morelos. México.

[2]Centro de Investigación en Biotecnología. Universidad Autónoma del Estado de Morelos. Cuernavaca, Morelos. México.

[3]School of Microbiology, University College Cork. Cork, Ireland.

[4]Environmental Research Institute, University College Cork. Cork, Ireland.

*Correspondence:
Dr. Ramón Alberto Batista-García
rabg@uaem.mx; rbatista25@yahoo.com

Keywords: metagenomic; virosphere; extreme environment; viral gene bioprospection; extremophile virome

Abstract

Viruses are the most abundant biological entities in the biosphere, and have the ability to infect Bacteria, Archaea and Eukaryotes. The virome is estimated to be at least ten times more abundant than the microbiome with 10^7 viruses per milliliter and 10^9 viral particles per gram in marine waters and sediments or soils, respectively. Viruses represent a largely unexplored genetic diversity, having an important role in the genomic plasticity of their hosts. Moreover, they also play a significant role in the dynamics of microbial populations. In recent years, metagenomic approaches have gained increasing popularity in the study of environmental viromes, offering the possibility of extending our knowledge related to both virus diversity and their functional characterization. Extreme environments represent an interesting source of both microbiota and their virome due to their particular physicochemical conditions, such as very high or very low temperatures and >1 atm hydrostatic pressures, among others. Despite the fact that some progress has been made in our understanding of the ecology of the microbiota in these habitats, few metagenomic studies have described the viromes present in extreme ecosystems. Thus, limited advances have been made in our understanding of the virus community structure in extremophilic ecosystems, as well as in their biotechnological potential. In this review, we critically analyse recent progress in metagenomic based approaches to explore the viromes in extreme environments and we discuss the potential for new discoveries, as well as methodological challenges and perspectives.

Table S1. Summary of studies metagenomics comparing to sequencing platforms, protocol extraction, and assembly and taxonomic tools

ID	Name of sample	ID sample	Number of reads or contigs	Type of sample	Country or Region	Type of sequencing	Method of extraction	Assembly	Taxonomic
SDEEP_ARCT_1a	EXPLODIVE	Arctic - Extra (2126)	27037	Sediment	Arctic Ocean	Pyrosequencing	CTAB/NaCl	CLC Genomics Workbench ver. 5.5	Megan
SDEEP_ATL_1a	EXPLODIVE	Atlantic - Extra (2125)	26826		Atlantic Ocean				
SDEEP_ATL_1b	EXPLODIVE	Atl_Vir (1157)	107090		Atlantic Ocean				
SDEEP_ARCT_1b	EXPLODIVE	Arct_Vir (1159)	63869		Arctic Ocean				
SDEEP_BKSEA_1a	EXPLODIVE	Black sea (1155)	784336		Black sea				
SDEEP_MED_1a	EXPLODIVE	Mediterrano (1161)	65340		Mediterraneo				
SDEEP_ATL_1c	EXPLODIVE	NE Atlanctic (1156)	165517		Atlantic Ocean				
SDEEP_ARCT_1c	EXPLODIVE	Arctic Ocean (1158)	79646		Arctic Ocean				
SDEEP_JAP_2a	(Hado)pelagic sediments	Izu-Ogasawara Trench (164)	46458		Izu-Ogasawara Trench Japon	Pyrosequencing	WI	WI	WI
SDEEP_JAP_2b	(Hado)pelagic sediments	Mariana Trench (165)	49584		Mariana Trench Japan				
SDEEP_JAP_2c	(Hado)pelagic sediments	off Shimokita Peninsula (166)	76498		Shimokita Peninsula Japon				
WDEEP_ITA_3a	Marine viromes	KM3 Ioinan Sea (3814)	9048	Deep sea-Seawater	Italy	Pyrosequencing	formamide/CTAB extraction	WI	Blast
WDEEP_CAN_4a	POV LineP transect	LJ12O (1394)	122565	Deep sea-Seawater	Canada	Pyrosequencing	WI	WI	WI
WDEEP_CAN_4b	POV LineP transect	LJ12D (1395)	49914						
WDEEP_CAN_4c	POV LineP transect	LA26O (1398)	70596						

WDEEP_CAN_4d	POV LineP transect	LA26D (1400)	68516						
WDEEP_CAN_4e	POV LineP transect	LF26O (1403)	147537						
WDEEP_CAN_4f	POV LineP transect	LF26D (1404)	125896						
WDEEP_CAN_4g	POV LineP transect	LJ26O (1407)	101179						
WDEEP_CAN_4h	POV LineP transect	LJ26D (1408)	55332						
WDEEP_CAN_4i	POV LineP transect	LJ4O (1414)	97126						
WDEEP_CAN_4j	POV LineP transect	LJ4D (1415)	98478						
WDEEP_EUA_4a	POV MBARI transect	M6O1K (1432)	225833		United States				
WDEEP_EUA_4b	POV MBARI transect	M7O4K (1433)	144588		United States				
OMZ_CAN_5a	Saanich Inlet	Saanich_200m_r200 (2915)	1728968	Oxygen minimum zones	Canada	Illumina	e Gentra Puregene Blood kit (Qiagen)	Geneious v7.1	Blastx
OMZ_CAN_5b	Saanich Inlet	Saanich_fosmids (3286)	47			Pyrosequencing			
OMZ_CHL_6a	2008OMZst3viral200m (898)	Pyrosequencing	96706		Chile	Chile	CTAB/ formamide	without assembly	Blastx
OMZ_CHL_6b	2008OMZst5viral200m (993)	Pyrosequencing	163531			Chile			
ARID_ATA_7a	Antarctic soil metaviromes	Antarctic hypolith reads (2726)	1057535	Hyperarid	Miers Valley; Antarctica	Illumina	WI	WI	WI
ARID_ATA_7b	Antarctic soil metaviromes	Antarctic open soil reads (2727)	870687		Miers Valley; Antarctica				
ARID_ATA_7c	Antarctic soil metaviromes	Antarctic hypolith contigs (2472)	32574		Miers Valley; Antarctica				
ARID_ATA_7d	Antarctic soil metaviromes	Antarctic open soil contigs (2473)	8442		Miers Valley; Antarctica				
ARID_NAM_8a	Namib Desert transect metaviromes	NAM_130 (5775)	3097		Namibia	Sanger	WI	WI	WI
ARID_NAM_8b	Namib Desert transect metaviromes	NAM_100 (5774)	2897			Sanger			
ARID_NAM_8c	Namib Desert transect metaviromes	NAM_70 (5773)	545			Sanger			
ARID_NAM_8d	Namib Desert transect metaviromes	NAM_40 ()							

ARID_NAM_8e	Namib hypolith 2012	Namib_hypolith_2012_contigs (2186)	4564			Illumina			
ARID_NAM_8f	Namib hypolith 2012	Namib_hypolith_2012_reads (2708)	841458			Illumina			
SALINE_NAM_9a	Archevir	P5_Ngallou (1700)	3018			Illumina			
SALINE_NAM_9b	Archevir	P2_Saloon(1701)	7302			Illumina			
SALINE_NAM_9c	Archevir	P6_Lake_Retba(1702)	884			Illumina			
SALINE_NAM_9d	Archevir	P7_Ngallou(1703)	1312		Namibia	Illumina		IDBA_UD	tBLASTx
SALINE_NAM_9e	Archevir	P8_Ngallou(1704)	3212			Illumina	QIAamp DNA mini kit (Qiagen)		
SALINE_NAM_9f	Archevir	P9_Ngallou(1705)	529			Illumina			
SALINE_EUA_10a	Saltern ponds	Saltern medium - 112805 (23)	39439						
SALINE_EUA_10b	Saltern ponds	Saltern medium - 111605 (24)	58319						
SALINE_EUA_10c	Saltern ponds	Saltern high - 111605 (25)	151180						
SALINE_EUA_10d	Saltern ponds	Saltern low - 0704 (26)	268049						
SALINE_EUA_10f	Saltern ponds	Saltern low - 111005 (27)	109836						
SALINE_EUA_10g	Saltern ponds	Saltern medium - 111005 (28)	39348		United_States	Pyrosequencing	Ultra Clean Soil DNA Kit	WI	
SALINE_EUA_10h	Saltern ponds	Saltern medium - 112205 (29)	55142						tBLASTx
SALINE_EUA_10i	Saltern ponds	Saltern high - 120705 (30)	46628						
SALINE_EUA_10j	Saltern ponds	Saltern high - 112805 (31)	4536						
SALINE_EUA_10k	Saltern ponds	Saltern low - 112805 (32)	62363	Hypersaline					
SALINE_EUA_11a	Freshwater	Tilapia Chanel 1105 (33)	264844				CTAB/ formamide		
SALINE_EUA_11b	Freshwater	Tilapia Channel 0406 (35)	60135		United_States	Pyrosequencing	Ultra Clean Soil DNA kit	WI	tBLASTx
SALINE_EUA_11c	Freshwater	Tilapia Channel 0806 (36)	56549						
SALINE_EUA_12a	Others	Tampa Bay induced(46)	279129		United_States	Pyrosequencing	WI	WI	WI

SALINE_EUA_12b	Others	Salton One (47)	55467						
SALINE_EUA_12c	Others	Salton Sea (46)	29814						
SALINE_EUA_12d	Others	Saltern Ponds fomids (1343)	42						
COLD_GRL_13a	Supraglacial	Cryoconite_viruses_curated (4314)	546	Cold Freshwater	Greenland icesheet plus Svalbard cryoconite	Illumina	QIAamp MinElute Virus Spin Kit	CLC Genomics Workbench	Qiime
COLD_ATA_14a	Lake limnopolar	Antartic Lake Spring	4132	Cold Freshwater	Antartic	Sanger	phenol-chloroform and ethanol precipitated.	WI	BLASTX
COLD_ATA_14b	Lake limnopolar	Antartic Lake Summer	387475	Cold Freshwater	Antartic	Sanger	phenol-chloroform and ethanol precipitated.	WI	BLASTX
HOT_EUA_15a	Yellowstone Hot Springs	Hot Spring Octopus (52)	22272	Hyperthermophile	United_States	Sanger	phenol-chloroform and ethanol precipitated.	ManSeq	BLASTx
HOT_EUA_15b	Yellowstone Hot Springs	Hot Spring Bear Paw (53)	8352	Hyperthermophile	United_States	Sanger			
HOT_TBV_16a	Brazos-Trinity Basin (IODP site 1320)	Brazos-Trinity 8mbsf (3813)	270730	Hyperthermophile	Mexico	Pyrosequencing	WI	WI	WI

*WI without information

Table S2. Structures of viral proteins derived from structural proteomic approaches or from the analysis of functionally unannotated open reading frames.

Family or order	Name	Extreme character	PDB ID	Molecule and function	Reference
Mimiviridae	*Megavirus chiliensis*	moderate salt	4XUL (XRAY)	Mg662 (GTP binding protein)	to be published
Mimiviridae	*Acanthamoeba polyphaga mimivirus*	moderate salt	4Z24, 5, 6 (XRAY)	R135 (outer layer protein, probably aryl alcohol oxidase)	Klose et al., 2015
Nimaviridae	White spot syndrome virus	moderate salt	2GJ2 (XRAY)<br>2GJI (NMR)	VP9 (hypothetical DNA binding protein)	Liu et al., 2006
Ligamenvirales	*Sulfolobus islandicus* rod-shaped virus 1	high temperature and low pH	2X5T, G, H, (XRAY)<br>2X3G (XRAY)<br>2X48 (XRAY)<br>2X4I (XRAY)	ORF131<br><br>ORF119<br>ORF55<br>ORF114a<br>(Scottish Structural Proteomics Facility)	Oke et al., 2010
Ligamenvirales	*Acidianus filamentous* virus 1	high temperature and low pH	3II2, 3, 3ILD, E (XRAY)	ORF157 (nuclease)	Goulet et al., 2010
Ligamenvirales	*Acidianus filamentous* virus 1	high temperature and low pH	3DF6, 3DJW (XRAY)	Afv1-99 (unknown)	Goulet et al., 2009a
Ligamenvirales	*Acidianus filamentous* virus 1	high temperature and low pH	2WB6 (XRAY)	Afv1-102 (unknown)	Keller et al., 2009
Ligamenvirales	*Sulfolobus islandicus* filamentous virus	high temperature and low pH	2H36 (XRAY)	ORF14 (putative structural protein)	Goulet et al., 2009b
Ligamenvirales	*Acidianus filamentous* virus 3	high temperature and low pH	2J6B, C (XRAY)	Afv3-109 (DNA binding protein)	Keller et al., 2007
Globuloviridae	*Pyrobaculum spherical virus*	high temperature	2X3M (XRAY)<br>2X5R (XRAY)<br>2X4J (XRAY)<br>2X5C (XRAY)<br>2VXZ (XRAY)	ORF239<br>ORF126<br>ORF137<br>ORF131<br>gp04<br>(Scottish Structural Proteomics Facility)	Oke et al., 2010
Fuselloviridae	*Sulfolobus spindle*-shape virus 1	high temperature and low pH	2VQC (XRAY)	F112 (hypothetical DNA binding protein)	Menon et al., 2008
Fuselloviridae	*Sulfolobus spindle*-	high temperature	1SKV (XRAY)	D-63 (hypothetical	Kraft et al., 2004

	shape virus 1	and low pH		adaptor protein)	
Bicaudaviridae	Acidianus two-tailed virus	high temperature and low pH	4ART (XRAY)<br>4ATS (XRAY)	ORF273 (novel fold)	Felisberto-Rodrigues et al., 2012
Bicaudaviridae	Acidianus two-tailed virus	high temperature and low pH	3FAJ (XRAY)	P131 (unknown)	to be published
satellite virus	*Sulfolobus islandicus* pSSVx	high temperature and low pH	3O27 (XRAY)	C68 (DNA binding protein)	Contursi et al., 2011

XRAY: obtained by X-ray crystallography; NMR: obtained by Nuclear Magnetic Resonance

References

Contursi, P., Farina, B., Pirone, L., Fusco, S., Russo, L., Bartolucci, S., Fattorusso, R., Pedone, E. (2014). Structural and functional studies of Stf76 from the *Sulfolobus islandicus* plasmid–virus pSSVx: a novel peculiar member of the winged helix–turn–helix transcription factor family. *Nucleic Acids Research*, 42(9), 5993-6011. doi:10.1093/nar/gku215

Felisberto-Rodrigues, C., Blangy, S., Goulet, A., Vestergaard, G., Cambillau, C., Garrett, R., Ortiz-Lombardía, M. (2012). Crystal Structure of ATVORF273, a New Fold for a Thermo- and Acido-Stable Protein from the *Acidianus* Two-Tailed Virus. *PLoS ONE*, 7(10), p.e45847. doi:10.1371/journal.pone.0045847.

Goulet, A., Lichiere, J., Prangishvili, D., van Tilbeurgh, H., Cambillau, C., Campanacci, V. (2010). Structure Of ORF157-K57A From *Acidianus filamentous* Virus 1. *Protein Data Bank, Rutgers University*. doi:10.2210/pdb3ild/pdb.

Goulet, A., Spinelli, S., Blangy, S., van Tilbeurgh, H., Leulliot, N., Basta, T., Prangishvili, D., Cambillau, C., Campanacci, V. (2009). The thermo- and acido-stable ORF-99 from the archaeal virus AFV1. *Protein Science*, 18(6), 1316-1320. doi:10.1002/pro.122.

Goulet, A., Spinelli, S., Blangy, S., van Tilbeurgh, H., Leulliot, N., Basta, T., Prangishvili, et al. (2009). The crystal structure of ORF14 from *Sulfolobus islandicus* filamentous virus. *Proteins: Structure, Function, and Bioinformatics*, 76(4), 1020-1022. doi:10.1002/prot.22448.

Keller, J., Leulliot, N., Cambillau, C., Campanacci, V., Porciero, S., Prangishvilli, D., Forterre, P., et al. (2007). Crystal structure of AFV3-109, a highly conserved protein from crenarchaeal viruses. *Virology Journal*, 4(1), 12. doi:10.1186/1743-422x-4-12.

Keller, J., Leulliot, N., Collinet, B., Campanacci, V., Cambillau, C., Pranghisvilli, D., van Tilbeurgh, H. (2009). Crystal structure of AFV1-102, a protein from the *Acidianus filamentous* Virus 1. *Protein Science*. NA-NA. doi:10.1002/pro.79.

Klose, T., Herbst, D., Zhu, H., Max, J., Kenttämaa, H., Rossmann, M. (2015). A Mimivirus Enzyme that Participates in Viral Entry. *Structure*, 23(6), 1058-1065. doi:10.1016/j.str.2015.03.023

Kraft, P., Kummel, D., Oeckinghaus, A., Gauss, G., Wiedenheft, B., Young, M., Lawrence, C. (2004). Structure of D-63 from *Sulfolobus* Spindle-Shaped Virus 1: Surface Properties of the Dimeric Four-Helix Bundle Suggest an Adaptor Protein Function. *Journal of Virology*, 78(14), 7438-7442.doi:10.1128/jvi.78.14.7438-7442.2004.

Liu, Y., Wu, J., Song, J., Sivaraman, J., Hew, C. (2006). Identification of a Novel Nonstructural Protein, VP9, from White Spot Syndrome Virus: Its Structure Reveals a Ferredoxin Fold with Specific Metal Binding Sites. *Journal of Virology*, 80(21), 10419-10427. doi:10.1128/jvi.00698-06.

Menon, S., Maaty, W., Corn, G., Kwok, S., Eilers, B., Kraft, P., Gillitzer, E., Young, M., Bothner, B., Lawrence, C. (2008). Cysteine usage in *Sulfolobus* spindle-shaped virus 1 and extension to hyperthermophilic viruses in general. *Virology*, 376(2), 270-278. doi:10.1016/j.virol.2008.03.026.

Oke, M., Carter, L., Johnson, K., Liu, H., McMahon, S., Yan, X., Kerou, M., et al. (2010). The Scottish Structural Proteomics Facility: targets, methods and outputs. *Journal of Structural and Functional Genomics*, 11(2), 167-180. doi:10.1007/s10969-010-9090-y.

Table S3. Structural proteins from extremophile viruses.

Family or order	Name	Extreme character	PDB ID	Molecule	Reference
Siphoviridae	marine siphovirus TW1	moderate salt	5WK1 (EM)	major capsid protein	Wang et al., 2018
Podoviridae	Procholococcus cyanophage P-SSP7	moderate salt	2XD8 (EM)	capsid	Liu et al., 2010
Inoviridae	Thermus phage PH75	high temperature	1HGV, Z, 1HH0 (FD)	capsid	Pederson et al., 2001
Sphaerolipoviridae	Thermus virus P23-77	high temperature	3ZMN, O, 3ZN4, 5, 6 (XRAY)	VP16 and VP17 capsid proteins	Rissanen et al., 2013
Turriviridae	Sulfolobus turreted icosahedral virus 1	high temperature and low pH	6BO3 (XRAY)	turret protein	to be published
Turriviridae	Sulfolobus turreted icosahedral virus 1	high temperature and low pH	3J31 (EM) 4IL7 (XRAY)	virus structural protein	Veesler et al., 2013
Turriviridae	Sulfolobus turreted icosahedral virus 1	high temperature and low pH	4IND (XRAY)	turret	to be published
Turriviridae	Sulfolobus turreted icosahedral virus 1	high temperature and low pH	3RKL (XRAY)	structural protein	to be published
Turriviridae	Sulfolobus turreted icosahedral virus 1	high temperature and low pH	2BBD (XRAY)	major capsid protein	Khayat et al., 2005
Nimaviridae	White spot syndrome virus	moderate salt	5HLJ (XRAY)	envelope protein VP24	Sun et al., 2016
Nimaviridae	White spot syndrome virus	moderate salt	2ED6, M (XRAY)	major envelope proteins VP26 and VP28	Tang et al., 2007
Ligamenvirales	Sulfolobus filamentous virus 1	high temperature and low pH	6D5F (EM)	virus	Liu et al., 2018
Ligamenvirales	Acidianus filamentous virus 1	high temperature and low pH	5W7G (EM)	viral envelope with lipid monolayer	Kasson et al., 2017
Ligamenvirales	Sulfolobus islandicus rod-shaped virus 2	high temperature and low pH	3J9X (EM)	virion	DiMaio et al., 2015
Ligamenvirales	Acidianus filamentous virus 1	high temperature and low pH	3FBL, Z (XRAY)	coat protein	Goulet et al., 2009
Liigamenvirales	Sulfolobus islandicus rod-shaped virus 1	high temperature and low pH	3F2E (XRAY)	coat protein	Szymczyna et al., 2009
Corticoviridae	Pseudoalteromonas	moderate salt	2W0C (XRAY)	virus	Abrescia et al., 2008

	phage PM2		2VVD (XRAY)<br>2VVE (XRAY)<br>2VVF (XRAY)	spike protein P1<br>spike protein P1<br>capsid protein P2	
Bicaudaviridae	Acidianus tailed spindle virus	high temperature and low pH	5EQW (XRAY)	major capsid protein	Hochstein et al., 2018
Lavidaviridae	Cafeteriavirus-dependent mavirus (virophage)	moderate salt	6G41, 2, 3, 4, 5 (XRAY)	major capsid protein, full length and truncated, penton protein	Born et al., 2018
Lavidaviridae	Sputnik virophage 3	moderate salt	3J26 (EM)	virus	Zhang et al., 2012
Satellite virus	Sputnik virophage	moderate salt	3KK5 (EM)	virus	Sun et al., 2009

XRAY: obtained by X-ray crystallography; NMR: obtained by Nuclear Magnetic Resonance; EM: obtained by cryo-electron microscopy; FD: obtained by fiber diffraction

References

Abrescia, N., Grimes, J., Kivelä, H., Assenberg, R., Sutton, G., Butcher, S., Bamford, J. et al. (2008). Insights into Virus Evolution and Membrane Biogenesis from the Structure of the Marine Lipid-Containing Bacteriophage PM2. *Molecular Cell*, 31(5), 749-761. doi:10.1016/j.molcel.2008.06.026.

Born, D., Reuter, L., Mersdorf, U., Mueller, M., Fischer, M., Meinhart, A., Reinstein, J. (2018). Capsid protein structure, self-assembly, and processing reveal morphogenesis of the marine virophage mavirus. *Proceedings of the National Academy of Sciences*, 115(28), 7332-7337. doi:10.1073/pnas.1805376115.

DiMaio, F., Yu, X., Rensen, E., Krupovic, M., Prangishvili, D. Egelman, E. (2015). A virus that infects a hyperthermophile encapsidates A-form DNA. *Science*, 348(6237), 914-917. doi:10.1126/science.aaa4181.

Goulet, A., Blangy, S., Redder, P., Prangishvili, D., Felisberto-Rodrigues, C., Forterre, P., Campanacci, V., Cambillau, C. (2009). *Acidianus filamentous* virus 1 coat proteins display a helical fold spanning the filamentous archaeal viruses lineage. *Proceedings of the National Academy of Sciences*, 106(50), 21155-21160. doi:10.1073/pnas.0909893106.

Hochstein, R., Bollschweiler, D., Dharmavaram, S., Lintner, N., Plitzko, J., Bruinsma, R., Engelhardt, H., Young, M., et al. (2018). Structural studies of *Acidianus* tailed spindle virus reveal a structural paradigm used in the assembly of spindle-shaped viruses. *Proceedings of the National Academy of Sciences*, 115(9), 2120-2125. doi:10.1073/pnas.1719180115.

Kasson, P., DiMaio, F., Yu, X., Lucas-Staat, S., Krupovic, M., Schouten, S., Prangishvili, D., Egelman, E. (2017). Model for a novel membrane envelope in a filamentous hyperthermophilic virus. *eLife*, 6. doi:10.7554/elife.26268.

Khayat, R., Tang, L., Larson, E., Lawrence, C., Young, M., Johnson, J. (2005). Structure of an archaeal virus capsid protein reveals a common ancestry to eukaryotic and bacterial viruses. *Proceedings of the National Academy of Sciences*, 102(52), 18944-18949. doi:10.1073/pnas.0506383102.

Liu, X., Zhang, Q., Murata, K., Baker, M., Sullivan, M., Fu, C., Dougherty, M., et al. (2010). Structural changes in a marine podovirus associated with release of its genome into *Prochlorococcus*. *Nature Structural & Molecular Biology*, 17(7), 830-836. doi:10.1038/nsmb.1823.

Liu, Y., Osinski, T., Wang, F., Krupovic, M., Schouten, S., Kasson, P., et al. (2018). Structural conservation in a membrane-enveloped filamentous virus infecting a hyperthermophilic acidophile. *Nature Communications*, 9(1). doi:10.1038/s41467-018-05684-6.

Pederson, D., Welsh, L., Marvin, D., Sampson, M., Perham, R., Yu, M., Slater, M. (2001). The protein capsid of filamentous bacteriophage PH75 from *Thermus thermophilus*. *Journal of Molecular Biology*, 309(2), 401-421. doi:10.1006/jmbi.2001.4685.

Rissanen, I., Grimes, J., Pawlowski, A., Mäntynen, S., Harlos, K., Bamford, J., Stuart, D. (2013). Bacteriophage P23-77 Capsid Protein Structures Reveal the Archetype of an Ancient Branch from a Major Virus Lineage. *Structure*, 21(5), 718-726. doi:10.1016/j.str.2013.02.026.

Sun, L., Su, Y., Zhao, Y., Fu, Z., Wu, Y. (2016). Crystal Structure of Major Envelope Protein VP24 from White Spot Syndrome Virus. *Scientific Reports*, 6(1). doi:10.1038/srep32309.

Sun, S., La Scola, B., Bowman, V., Ryan, C., Whitelegge, J., Raoult, D., Rossmann, M. (2009). Structural Studies of the Sputnik Virophage. *Journal of Virology*, 84(2), 894-897. doi: 10.1128/JVI.01957-09

Szymczyna, B., Taurog, R., Young, M., Snyder, J., Johnson, J. and Williamson, J. (2009). Synergy of NMR, Computation, and X-Ray Crystallography for Structural Biology. *Structure*, 17(4), 499-507. doi:10.1016/j.str.2009.03.001.

Tang, X., Wu, J., Sivaraman, J., Hew, C. (2007). Crystal Structures of Major Envelope Proteins VP26 and VP28 from White Spot Syndrome Virus Shed Light on Their Evolutionary Relationship. *Journal of Virology*, 81(12), 6709-6717. doi:10.1128/jvi.02505-06.

Veesler, D., Ng, T., Sendamarai, A., Eilers, B., Lawrence, C., Lok, S., Young, M., Johnson, J., Fu, C. (2013). Atomic structure of the 75 MDa extremophile *Sulfolobus turreted* icosahedral virus determined by CryoEM and X-ray crystallography. *Proceedings of the National Academy of Sciences*, 110(14), 504-5509. doi:10.1073/pnas.1300601110.

Wang, Z., Hardies, S., Fokine, A., Klose, T., Jiang, W., Cho, B., Rossmann, M. (2018). Structure of the Marine Siphovirus TW1: Evolution of Capsid-Stabilizing Proteins and Tail Spikes. *Structure*, 26(2), 238-248.e3. doi:10.1016/j.str.2017.12.001.

Table S4. DNA binding proteins and proteins that bind histones from extremophilic viruses.

Family or order	Name	Extreme character	PDB ID	molecule	Reference
Iridoviridae	Singapore grouper iridovirus	moderate salt	3RJ2 (XRAY)	H3 binding protein	Tran et al., 2011
Turriviridae	Sulfolobus turreted icosahedral virus 1	high temperature and low pH	2J85 (XRAY)	DNA binding protein	Larson et al., 2007
Turriviridae	Sulfolobus turreted icosahedral virus 1	high temperature and low pH	2CO5 (XRAY)	DNA binding protein	Larson et al., 2007a
Nimaviridae	White spot syndrome virus	moderate salt	2ZUG (XRAY)	histone-binding DNA-like protein	Wang et al., 2008
Ligamenvirales	Acidianus filamentous virus 1	high temperature and low pH	2LVH (NMR)	DNA binding protein (Zn finger)	Guilliere et al., 2013
Ligamenvirales	Acidianus filamentous virus 6	high temperature and low pH	4HV0 (XRAY)	transcription regulator	Peixeiro et al., 2013
Ligamenvirales	Sulfolobus islandicus rod-shaped virus 1	high temperature and low pH	2KEL (NMR)	transcriptional regulator SvtR	Guilliere et al., 2009
Fuselloviridae	Sulfolobus spindle-shape virus 1	high temperature and low pH	4LID (XRAY)	DNA binding protein	to be published
Fuselloviridae	Sulfolobus islandicus plasmid-virus pSSVx	high temperature and low pH	2MLG (NMR)	transcription factor Stf76	Contursi et al., 2014
Fuselloviridae	Sulfolobus virus Ragged Hills	high temperature and low pH	4AAI (NMR)	DNA binding protein E73	Schlenker et al., 2012
Fuselloviridae	Sulfolobus spindle-shape virus 1	high temperature and low pH	2WBT (XRAY)	zinc finger protein	to be published
Fuselloviridae	Sulfolobus spindle-shape virus 1	high temperature and low pH	1TBX (XRAY)	DNA binding protein F-93	Kraft et al., 2004

XRAY: obtained by X-ray crystallography; NMR: obtained by Nuclear Magnetic Resonance

References

Contursi, P., Farina, B., Pirone, L., Fusco, S., Russo, L., Bartolucci, S., Fattorusso, R., Pedone, E. (2014). Structural and functional studies of Stf76 from the *Sulfolobus islandicus* plasmid–virus pSSVx: a novel peculiar member of the winged helix–turn–helix transcription factor family. *Nucleic Acids Research*, 42(9), 5993-6011 doi:10.1093/nar/gku215.

Guillière, F., Danioux, C., Jaubert, C., Desnoues, N., Delepierre, M., Prangishvili, D., Sezonov, G., Guijarro, J. (2013). Solution Structure of an Archaeal DNA Binding Protein with an Eukaryotic Zinc Finger Fold. *PLoS ONE*, 8(1), p.e52908. doi:10.1371/journal.pone.0052908.

Guillière, F., Peixeiro, N., Kessler, A., Raynal, B., Desnoues, N., Keller, J., Delepierre, M., Prangishvili, D., et al. (2009). Structure, Function, and Targets of the Transcriptional Regulator SvtR from the Hyperthermophilic Archaeal Virus SIRV1. *Journal of Biological Chemistry*, 284(33), 22222-22237. doi: 10.1074/jbc.M109.029850

Kraft, P., Oeckinghaus, A., Kummel, D., Gauss, G., Gilmore, J., Wiedenheft, B., Young, M., Lawrence, C. (2004). Crystal Structure of F-93 from *Sulfolobus* Spindle-Shaped Virus 1, a Winged-Helix DNA Binding Protein. *Journal of Virology*, 78(21), 11544-11550.doi:10.1128/jvi.78.21.11544-11550.2004.

Larson, E., Eilers, B., Menon, S., Reiter, D., Ortmann, A., Young, M., Lawrence, C. (2007). A winged-helix protein from *Sulfolobus turreted* icosahedral virus points toward stabilizing disulfide bonds in the intracellular proteins of a hyperthermophilic virus. *Virology*, 368(2), 249-261. doi.org/10.1016/j.virol.2007.06.040

Larson, E., Eilers, B., Reiter, D., Ortmann, A., Young., Lawrence, C. (2007). A new DNA binding protein highly conserved in diverse crenarchaeal viruses. *Virology*, 363(2), 387-396 doi:10.1016/j.virol.2007.01.027.

Peixeiro, N., Keller, J., Collinet, B., Leulliot, N., Campanacci, V., Cortez, D., Cambillau, C., Nitta, K., et al. (2012). Structure and Function of AvtR, a Novel Transcriptional Regulator from a Hyperthermophilic Archaeal Lipothrixvirus. *Journal of Virology*, 87(1), 124-136. doi:10.1128/jvi.01306-12.

Schlenker, C., Goel, A., Tripet, B., Menon, S., Willi, T., Dlakić, M., et al. (2012). Structural Studies of E73 from a Hyperthermophilic Archaeal Virus Identify the “RH3” Domain, an Elaborated Ribbon–Helix–Helix Motif Involved in DNA Recognition. *Biochemistry*, 51(13), 2899-2910. doi:10.1021/bi201791s.

Tran, B., Chen, L., Liu, Y., Wu, J., Velazquez-Campoy, A., Sivaraman, J. Hew, C. (2011). Novel Histone H3 Binding Protein ORF158L from the Singapore Grouper Iridovirus. *Journal of Virology*, 85(17), 9159-9166. doi:10.1128/jvi.02219-10.

Wang, H., Wang, H., Ko, T., Lee, Y., Leu, J., Ho, C., Huang, W., Lo, C. and Wang, A. (2008). White spot syndrome virus protein ICP11: A histone-binding DNA mimic that disrupts nucleosome assembly. *Proceedings of the National Academy of Sciences*, 105(52), 20758-20763. doi:10.1073/pnas.0811233106.

Table S5. Enzymes from extremophilic viruses.

Family or order	Name	Environment	PDB ID	Molecule	Reference
Caudovirales	Thermus phage G20c	high temperature	6IBG (XRAY)	portal protein	Bayfield et al., 2019
Myoviridae	Prochlorococcus phage P-HM1	moderate salt	5HI8 (XRAY)	phycobiliprotein lyase	Gasper et al., 2017
Myoviridae	cyanophage S-TIM5	moderate salt	4P9C, D, E (XRAY)	deoxycytidylate deaminase	Marx and Alian 2014
Myoviridae	Synechococcus phage S-SSM7	moderate salt	3UWA, B (XRAY)	peptide deformylase	Frank et al., 2013
Phycodnaviridae	Ostreococcus tauri virus 2	moderate salt, high copper	4B8N (XRAY)	cytochrome b5	Reid et al., 2013
Mimiviridae	Acanthamoeba polyphaga mimivirus	moderate salt	5YEU, T (XRAY)	Cas4-like protein	Dou et al., 2018
Mimiviridae	Acanthamoeba polyphaga mimivirus	moderate salt	6AX6, 7 (XRAY)	lysyl hydroxylase	Guo et al., 2018
Mimiviridae	Acanthamoeba polyphaga mimivirus	moderate salt	5XC3, 5 (XRAY)	Rab GTPase	Ku et al., 2017
Mimiviridae	Acanthamoeba polyphaga mimivirus	moderate salt	5X55 (XRAY)	uracil-DNA glycosylase	Kwon et al., 2017
Mimiviridae	Megavirus chiliensis	moderate salt	4P37, 4WSE (XRAY)	polyadenylate synthase	Priet et al., 2015
Mimiviridae	Megavirus chiliensis	moderate salt	4U4I (XRAY)	superoxide dismutase	Lartigue et al., 2014
Mimiviridae	Megavirus chiliensis	moderate salt	4TQG (XRAY)	UDP-GlcNac 4,6-dehydratase 5-epimerase	Piacente et al., 2014
Mimiviridae	Acanthamoeba polyphaga mimivirus	moderate salt	4NRW (XRAY)	DNA glycosylase Nei1-G86D	Prakash et al., 2014
Mimiviridae	Acanthamoeba	moderate salt	4MB7 (XRAY)	DNA glycosylase	Prakash et al., 2013

	polyphaga mimivirus			Nei2	
Mimiviridae	Acanthamoeba polyphaga mimivirus	moderate salt	4AMQ (XRAY)	nucleotidyl transferase	to be published
Mimiviridae	Megavirus chiliensis	moderate salt	4AMS (XRAY)	nucleotidyl transferase	to be published
Mimiviridae	Acanthamoeba polyphaga mimivirus	moderate salt	3TD7 (XRAY)	sulfhydryl oxidase	Hakim et al., 2012
Mimiviridae	Acanthamoeba polyphaga mimivirus	moderate salt	3VK7, 8 (XRAY)	DNA glycosylase Nei1	Imamura et al., 2012
Mimiviridae	Acanthamoeba polyphaga mimivirus	moderate salt	3DKD, 3GPA, 3GP9, 3G2X, 3FCW, 3FCV, 3FC9, 3FBF, 3FBE, 3FBC, 3FBB, 3EVW, 3EVO, 3EVM, 3ETM, 3ENA, 3EMT, 3EM1, 3ELH, 3EJM, 3EIC, 3EE3, 3DDI, 3B6B, 2B8Q, 2B8P (XRAY)	nucleoside diphosphate kinase, wild type and mutants	Jeudy et al., 2009
Mimiviridae	Acanthamoeba polyphaga mimivirus	moderate salt	3A42, 5, 6 (XRAY)	DNA glycosylase Nei1	Imamura et al., 2009
Mimiviridae	Acanthamoeba polyphaga mimivirus	moderate salt	3GWN (XRAY)	sulfhydryl oxidase	Hakim and Fass, 2009
Iridoviridae	grouper iridovirus	moderate salt	5VMN, O (XRAY)	Bcl-2 protein apoptosis inhibitor	Banjara et al., 2018
Iridoviridae	grouper iridovirus	moderate salt	3KHS (XRAY)	purine nucleoside	Kang et al., 2010

				phosphorylase	
Turriviridae	Sulfolobus turreted icosahedral virus 1	high temperature and low pH	4R2H, I (XRAY)	DNA packing ATPase B204	Dellas et al., 2015
Turriviridae	Sulfolobus turreted icosahedral virus 2	high temperature and low pH	4KFR, S, T, U (XRAY)	DNA packing ATPase B204	Happonen et al., 2013
Turriviridae	Sulfolobus turreted icosahedral virus 1	high temperature and low pH	2C0N (XRAY)	glycosyltransferase	Larson et al., 2006
nimaviridae	White spot syndrome virus	moderate salt	5Y5O, P, Q (XRAY)	dUTPase	Zang et al., 2018
ligamenvirales	Sulfolobus islandicus rudivirus 3	high temperature and low pH	6EXP (XRAY)	anti-CRISPR protein	He et al., 2018
ligamenvirales	Acidianus filamentous virus 1	high temperature and low pH	3II2, 3, 3ILD, E (XRAY)	nuclease ORF157	Goulet et al., 2010
fuselloviridae	Sulfolobus spindle-shape virus 1	high temperature and low pH	3VCF, 4DKS (XRAY)	integrase	Zhan et al., 2012
fuselloviridae	Sulfolobus spindle-shape virus 1	high temperature and low pH	3UXU (XRAY)	integrase	Eilers et al., 2012
fuselloviridae	Sulfolobus virus Ragged Hills	high temperature and low pH	2W8M (XRAY)	PD-(D/E)XK nuclease	Menon et al., 2010

XRAY: obtained by X-ray crystallography

References

Banjara, S., Mao, J., Ryan, T., Caria, S., Kvansakul, M. (2018). Grouper iridovirus GIV66 is a Bcl-2 protein that inhibits apoptosis by exclusively sequestering Bim. *Journal of Biological Chemistry*, 293(15), 5464-5477. doi:10.1074/jbc.ra117.000591.

Bayfield, O., Klimuk, E., Winkler, D., Hesketh, E., Chechik, M., Cheng, N., Dykeman, E., et al. (2019). Cryo-EM structure and in vitro DNA packaging of a thermophilic virus with supersized T=7 capsids. *Proceedings of the National Academy of Sciences*, 116(9), 3556-3561 doi: 10.1073/pnas.1813204116

Dellas, N., Snyder, J., Dills, M., Nicolay, S., Kerchner, K., Brumfield, S., Lawrence, C., Young, M. (2015). Structure-Based Mutagenesis of Sulfolobus Turreted Icosahedral Virus B204 Reveals Essential Residues in the Virion-Associated DNA-Packaging ATPase. *Journal of Virology*, 90(6), 2729-2739. doi:10.1128/jvi.02435-15.
Dou, C., Yu, M., Gu, Y., Wang, J., Yin, K., Nie, C., Zhu, X., Qi, S., Wei, Y., Cheng, W. (2018). Structural and Mechanistic Analyses Reveal a Unique Cas4-like Protein in the Mimivirus Virophage Resistance Element System. *Science*, 3, 1-10. doi:10.1016/j.isci.2018.04.001.

Eilers, B., Young, M., Lawrence, C. (2012). The Structure of an Archaeal Viral Integrase Reveals an Evolutionarily Conserved Catalytic Core yet Supports a Mechanism of DNA Cleavage in trans. *Journal of Virology*, 86(15), 8309-8313. doi:10.1128/jvi.00547-12.

Frank, J., Lorimer, D., Youle, M., Witte, P., Craig, T., Abendroth, J., Rohwer, F., Edwards, R., Segall, A.,Burgin, A. (2013). Structure and function of a cyanophage-encoded peptide deformylase. *The ISME Journal*, 7(6), 1150-1160. doi:10.1038/ismej.2013.4.

Frank, J., Lorimer, D., Youle, M., Witte, P., Craig, T., Abendroth, J., Rohwer, F., et al. (2013). Structure and function of a cyanophage-encoded peptide deformylase. *The ISME Journal*, 7(6), 1150-1160. doi: 10.1007/s00705-017-3510-2

Gasper, R., Schwach, J., Hartmann, J., Holtkamp, A., Wiethaus, J., Riedel, N., Hofmann, E., Frankenberg-Dinkel, N. (2017). Distinct Features of Cyanophage-encoded T-type Phycobiliprotein Lyase ΦCpeT. *Journal of Biological Chemistry*, 292(8), 3089-3098. doi:10.1074/jbc.m116.769703

Goulet, A., Pina, M., Redder, P., Prangishvili, D., Vera, L., Lichiere, J., Leulliot, N., et al. (2010). ORF157 from the Archaeal Virus Acidianus Filamentous Virus 1 Defines a New Class of Nuclease. *Journal of Virology*, 84(10), 5025-5031. doi:10.1128/jvi.01664-09.

Guo, H., Tsai, C., Terajima, M., Tan, X., Banerjee, P., Miller, M., Liu, X., Yu, J., Byemerwa, J., et al. (2018). Pro-metastatic collagen lysyl hydroxylase dimer assemblies stabilized by Fe2+-binding. *Nature Communications*, 9(1). doi:10.1038/s41467-018-02859-z.

Hakim, M., Ezerina, D., Alon, A., Vonshak, O., Fass, D. (2012). Exploring ORFan Domains in Giant Viruses: Structure of Mimivirus Sulfhydryl Oxidase R596. *PLoS ONE*, 7(11), p.e50649. doi:10.1371/journal.pone.0050649.

Happonen, L., Oksanen, E., Liljeroos, L., Goldman, A., Kajander, T., Butcher, S. (2013). The Structure of the NTPase That Powers DNA Packaging into Sulfolobus Turreted Icosahedral Virus 2. *Journal of Virology*, 87(15). 8388-8398. doi:10.1128/jvi.00831-13.

He, F., Bhoobalan-Chitty, Y., Van, L., Kjeldsen, A., Dedola, M., Makarova, K., Koonin, E., Brodersen, D. et al, (2018). Anti-CRISPR proteins encoded by archaeal lytic viruses inhibit subtype I-D immunity. *Nature Microbiology*, 3(4), 461-469. doi:10.1038/s41564-018-0120-z.

Imamura, K., Averill, A., Wallace, S., Doublié, S. (2011). Structural Characterization of Viral Ortholog of Human DNA Glycosylase NEIL1 Bound to Thymine Glycol or 5-Hydroxyuracil-containing DNA. *Journal of Biological Chemistry*, 287(6).4288-4298. doi:10.1074/jbc.m111.315309.

Jeudy, S., Lartigue, A., Claverie, J., Abergel, C. (2009). Dissecting the Unique Nucleotide Specificity of Mimivirus Nucleoside Diphosphate Kinase. *Journal of Virology*, 83(14). 7142-7150. doi:10.1128/jvi.00511-09

Kang, Y., Zhang, Y., Allan, P., Parker, W., Ting, J., Chang, C., Ealick, S. (2010). Structure of grouper iridovirus purine nucleoside phosphorylase. *Acta Crystallographica Section D Biological Crystallography*, 66(2).155-162. doi:10.1107/s0907444909048276.

Ku, B., You, J., Oh, K., Yun, H., Lee, H., Shin, H., Jung, J., Shin, Y., Kim, S. (2017). Crystal structures of two forms of the Acanthamoeba polyphaga mimivirus Rab GTPase. *Archives of Virology*, 162(11). 3407-3416. doi:10.1007/s00705-017-3510-2.

Ku, B., You, J., Oh, K., Yun, H., Lee, H., Shin, H., Jung, J., Shin, Y., Kim, S. (2017). Crystal structures of two forms of the Acanthamoeba polyphaga mimivirus Rab GTPase. *Archives of Virology*, 162(11), 3407-3416.

Kwon, E., Pathak, D., Chang, H. and Kim, D. (2017). Crystal structure of mimivirus uracil-DNA glycosylase. *PLOS ONE*, 12(8),0182382. doi:10.1371/journal.pone.0182382.

Larson, E., Reiter, D., Young, M., Lawrence, C. (2006). Structure of A197 from Sulfolobus Turreted Icosahedral Virus: a Crenarchaeal Viral Glycosyltransferase Exhibiting the GT-A Fold. *Journal of Virology*, 80(15).7636-7644. doi:10.1128/jvi.00567-06.

Lartigue, A., Burlat, B., Coutard, B., Chaspoul, F., Claverie, J., Abergel, C. (2014). The Megavirus Chilensis Cu,Zn-Superoxide Dismutase: the First Viral Structure of a Typical Cellular Copper Chaperone-Independent Hyperstable Dimeric Enzyme. *Journal of Virology*, 89(1),824-832. doi:10.1128/jvi.02588-14.

Marx, A., Alian, A. (2014). The First Crystal Structure of a dTTP-bound Deoxycytidylate Deaminase Validates and Details the Allosteric-Inhibitor Binding Site. *Journal of Biological Chemistry*, 290(1). 682-690. doi:10.1074/jbc.m114.617720.

Menon, S., Eilers, B., Young, M.,Lawrence, C. (2010). The Crystal Structure of D212 from Sulfolobus Spindle-Shaped Virus Ragged Hills Reveals a New Member of the PD-(D/E)XK Nuclease Superfamily. *Journal of Virology*, 84(12). 5890-5897. doi:10.1128/jvi.01663-09.

Piacente, F., De Castro, C., Jeudy, S., Molinaro, A., Salis, A., Damonte, G., Bernardi, C., et al. (2014). Giant VirusMegavirus chilensisEncodes the Biosynthetic Pathway for Uncommon Acetamido Sugars. *Journal of Biological Chemistry*, 289(35), 24428-24439.doi:10.1074/jbc.m114.588947.

Prakash, A., Carroll, B., Sweasy, J., Wallace, S., Doublié, S. (2014). Genome and cancer single nucleotide polymorphisms of the human NEIL1 DNA glycosylase: Activity, structure, and the effect of editing. *DNA Repair*, 14,17-26. doi:10.1016/j.dnarep.2013.12.003.

Priet, S., Lartigue, A., Debart, F., Claverie, J. Abergel, C. (2015). mRNA maturation in giant viruses: variation on a theme. *Nucleic Acids Research*, 43(7). 3776-3788. doi:10.1093/nar/gkv224.

Reid, E., Weynberg, K., Love, J., Isupov, M., Littlechild, J., Wilson, W., Kelly, S., Lamb, D., Allen, M. (2013). Functional and structural characterisation of a viral cytochromeb5. *FEBS Letters*, 587(22).3633-3639. doi:10.1016/j.febslet.2013.09.035.

Zang, K., Li, F., Ma, Q. (2017). The dUTPase of white spot syndrome virus assembles its active sites in a noncanonical manner. *Journal of Biological Chemistry*, 293(3),1088-1099. doi:10.1074/jbc.m117.815266.

Zhan, Z., Ouyang, S., Liang, W., Zhang, Z., Liu, Z., Huang, L. (2012). Structural and functional characterization of the C-terminal catalytic domain of SSV1 integrase. *Acta Crystallographica Section D Biological Crystallography*, 68(6).659-670. doi:10.1107/s0907444912007202.
